# Genetic and non-genetic factors affecting the expression of COVID-19-relevant genes in the large airway epithelium

**DOI:** 10.1186/s13073-021-00866-2

**Published:** 2021-04-21

**Authors:** Silva Kasela, Victor E. Ortega, Molly Martorella, Suresh Garudadri, Jenna Nguyen, Elizabeth Ampleford, Anu Pasanen, Srilaxmi Nerella, Kristina L. Buschur, Igor Z. Barjaktarevic, R. Graham Barr, Eugene R. Bleecker, Russell P. Bowler, Alejandro P. Comellas, Christopher B. Cooper, David J. Couper, Gerard J. Criner, Jeffrey L. Curtis, MeiLan K. Han, Nadia N. Hansel, Eric A. Hoffman, Robert J. Kaner, Jerry A. Krishnan, Fernando J. Martinez, Merry-Lynn N. McDonald, Deborah A. Meyers, Robert Paine, Stephen P. Peters, Mario Castro, Loren C. Denlinger, Serpil C. Erzurum, John V. Fahy, Elliot Israel, Nizar N. Jarjour, Bruce D. Levy, Xingnan Li, Wendy C. Moore, Sally E. Wenzel, Joe Zein, NHLBI SubPopulations and InteRmediate Outcome Measures In COPD Study (SPIROMICS), Neil E. Alexis, Wayne H. Anderson, Mehrdad Arjomandi, Igor Barjaktarevic, R. Graham Barr, Patricia Basta, Lori A. Bateman, Surya P. Bhatt, Eugene R. Bleecker, Richard C. Boucher, Russell P. Bowler, Stephanie A. Christenson, Alejandro P. Comellas, Christopher B. Cooper, David J. Couper, Gerard J. Criner, Ronald G. Crystal, Jeffrey L. Curtis, Claire M. Doerschuk, Mark T. Dransfield, Brad Drummond, Christine M. Freeman, Craig Galban, Mei Lan K. Han, Nadia N. Hansel, Annette T. Hastie, Eric A. Hoffman, Yvonne Huang, Robert J. Kaner, Richard E. Kanner, Eric C. Kleerup, Jerry A. Krishnan, Lisa M. LaVange, Stephen C. Lazarus, Fernando J. Martinez, Deborah A. Meyers, Wendy C. Moore, John D. Newell, Robert Paine, Laura Paulin, Stephen P. Peters, Cheryl Pirozzi, Nirupama Putcha, Elizabeth C. Oelsner, Wanda K. O’Neal, Victor E. Ortega, Sanjeev Raman, Stephen I. Rennard, Donald P. Tashkin, J. Michael Wells, Robert A. Wise, Prescott G. Woodruff, NHLBI Trans-Omics for Precision Medicine (TOPMed) Consortium, Namiko Abe, Gonçalo Abecasis, Francois Aguet, Christine Albert, Laura Almasy, Alvaro Alonso, Seth Ament, Peter Anderson, Pramod Anugu, Deborah Applebaum-Bowden, Kristin Ardlie, Dan Arking, Donna K. Arnett, Allison Ashley-Koch, Stella Aslibekyan, Tim Assimes, Paul Auer, Dimitrios Avramopoulos, John Barnard, Kathleen Barnes, R. Graham Barr, Emily Barron-Casella, Lucas Barwick, Terri Beaty, Gerald Beck, Diane Becker, Lewis Becker, Rebecca Beer, Amber Beitelshees, Emelia Benjamin, Takis Benos, Marcos Bezerra, Larry Bielak, Joshua Bis, Thomas Blackwell, John Blangero, Eric Boerwinkle, Donald W. Bowden, Russell Bowler, Jennifer Brody, Ulrich Broeckel, Jai Broome, Karen Bunting, Esteban Burchard, Carlos Bustamante, Erin Buth, Brian Cade, Jonathan Cardwell, Vincent Carey, Cara Carty, Richard Casaburi, James Casella, Peter Castaldi, Mark Chaffin, Christy Chang, Yi-Cheng Chang, Daniel Chasman, Sameer Chavan, Bo-Juen Chen, Wei-Min Chen, Yii-Der Ida Chen, Michael Cho, Seung Hoan Choi, Lee-Ming Chuang, Mina Chung, Ren-Hua Chung, Clary Clish, Suzy Comhair, Matthew Conomos, Elaine Cornell, Adolfo Correa, Carolyn Crandall, James Crapo, L. Adrienne Cupples, Joanne Curran, Jeffrey Curtis, Brian Custer, Coleen Damcott, Dawood Darbar, Sayantan Das, Sean David, Colleen Davis, Michelle Daya, Mariza de Andrade, Lisa de las Fuentes, Michael DeBaun, Ranjan Deka, Dawn DeMeo, Scott Devine, Qing Duan, Ravi Duggirala, Jon Peter Durda, Susan Dutcher, Charles Eaton, Lynette Ekunwe, Adel El Boueiz, Patrick Ellinor, Leslie Emery, Serpil Erzurum, Charles Farber, Tasha Fingerlin, Matthew Flickinger, Myriam Fornage, Nora Franceschini, Chris Frazar, Mao Fu, Stephanie M. Fullerton, Lucinda Fulton, Stacey Gabriel, Weiniu Gan, Shanshan Gao, Yan Gao, Margery Gass, Bruce Gelb, Xiaoqi ( Priscilla) Geng, Mark Geraci, Soren Germer, Robert Gerszten, Auyon Ghosh, Richard Gibbs, Chris Gignoux, Mark Gladwin, David Glahn, Stephanie Gogarten, Da-Wei Gong, Harald Goring, Sharon Graw, Daniel Grine, C. Charles Gu, Yue Guan, Xiuqing Guo, Namrata Gupta, Jeff Haessler, Michael Hall, Daniel Harris, Nicola L. Hawley, Jiang He, Ben Heavner, Susan Heckbert, Ryan Hernandez, David Herrington, Craig Hersh, Bertha Hidalgo, James Hixson, Brian Hobbs, John Hokanson, Elliott Hong, Karin Hoth, Chao ( Agnes) Hsiung, Yi-Jen Hung, Haley Huston, Chii Min Hwu, Marguerite Ryan Irvin, Rebecca Jackson, Deepti Jain, Cashell Jaquish, Min A. Jhun, Jill Johnsen, Andrew Johnson, Craig Johnson, Rich Johnston, Kimberly Jones, Hyun Min Kang, Robert Kaplan, Sharon Kardia, Sekar Kathiresan, Shannon Kelly, Eimear Kenny, Michael Kessler, Alyna Khan, Wonji Kim, Greg Kinney, Barbara Konkle, Charles Kooperberg, Holly Kramer, Christoph Lange, Ethan Lange, Leslie Lange, Cathy Laurie, Cecelia Laurie, Meryl LeBoff, Jiwon Lee, Seunggeun Shawn Lee, Wen-Jane Lee, Jonathon LeFaive, David Levine, Dan Levy, Joshua Lewis, Xiaohui Li, Yun Li, Henry Lin, Honghuang Lin, Keng Han Lin, Xihong Lin, Simin Liu, Yongmei Liu, Yu Liu, Ruth J. F. Loos, Steven Lubitz, Kathryn Lunetta, James Luo, Michael Mahaney, Barry Make, Ani Manichaikul, Jo Ann Manson, Lauren Margolin, Lisa Martin, Susan Mathai, Rasika Mathias, Susanne May, Patrick McArdle, Merry-Lynn McDonald, Sean McFarland, Stephen McGarvey, Daniel McGoldrick, Caitlin McHugh, Hao Mei, Luisa Mestroni, Deborah A. Meyers, Julie Mikulla, Nancy Min, Mollie Minear, Ryan L. Minster, Braxton D. Mitchell, Matt Moll, May E. Montasser, Courtney Montgomery, Arden Moscati, Solomon Musani, Stanford Mwasongwe, Josyf C. Mychaleckyj, Girish Nadkarni, Rakhi Naik, Take Naseri, Pradeep Natarajan, Sergei Nekhai, Sarah C. Nelson, Bonnie Neltner, Deborah Nickerson, Kari North, Jeff O’Connell, Tim O’Connor, Heather Ochs-Balcom, David Paik, Nicholette Palmer, James Pankow, George Papanicolaou, Afshin Parsa, Juan Manuel Peralta, Marco Perez, James Perry, Ulrike Peters, Patricia Peyser, Lawrence S. Phillips, Toni Pollin, Wendy Post, Julia Powers Becker, Meher Preethi Boorgula, Michael Preuss, Bruce Psaty, Pankaj Qasba, Dandi Qiao, Zhaohui Qin, Nicholas Rafaels, Laura Raffield, Vasan S. Ramachandran, D. C. Rao, Laura Rasmussen-Torvik, Aakrosh Ratan, Susan Redline, Robert Reed, Elizabeth Regan, Alex Reiner, Muagututi‘a Sefuiva Reupena, Ken Rice, Stephen Rich, Dan Roden, Carolina Roselli, Jerome Rotter, Ingo Ruczinski, Pamela Russell, Sarah Ruuska, Kathleen Ryan, Ester Cerdeira Sabino, Danish Saleheen, Shabnam Salimi, Steven Salzberg, Kevin Sandow, Vijay G. Sankaran, Christopher Scheller, Ellen Schmidt, Karen Schwander, David Schwartz, Frank Sciurba, Christine Seidman, Jonathan Seidman, Vivien Sheehan, Stephanie L. Sherman, Amol Shetty, Aniket Shetty, Wayne Hui-Heng Sheu, M. Benjamin Shoemaker, Brian Silver, Edwin Silverman, Jennifer Smith, Josh Smith, Nicholas Smith, Tanja Smith, Sylvia Smoller, Beverly Snively, Michael Snyder, Tamar Sofer, Nona Sotoodehnia, Adrienne M. Stilp, Garrett Storm, Elizabeth Streeten, Jessica Lasky Su, Yun Ju Sung, Jody Sylvia, Adam Szpiro, Carole Sztalryd, Daniel Taliun, Hua Tang, Margaret Taub, Kent D. Taylor, Matthew Taylor, Simeon Taylor, Marilyn Telen, Timothy A. Thornton, Machiko Threlkeld, Lesley Tinker, David Tirschwell, Sarah Tishkoff, Hemant Tiwari, Catherine Tong, Russell Tracy, Michael Tsai, Dhananjay Vaidya, David Van Den Berg, Peter VandeHaar, Scott Vrieze, Tarik Walker, Robert Wallace, Avram Walts, Fei Fei Wang, Heming Wang, Karol Watson, Daniel E. Weeks, Bruce Weir, Scott Weiss, Lu Chen Weng, Jennifer Wessel, Cristen Willer, Kayleen Williams, L. Keoki Williams, Carla Wilson, James Wilson, Quenna Wong, Joseph Wu, Huichun Xu, Lisa Yanek, Ivana Yang, Rongze Yang, Norann Zaghloul, Maryam Zekavat, Yingze Zhang, Snow Xueyan Zhao, Wei Zhao, Degui Zhi, Xiang Zhou, Xiaofeng Zhu, Michael Zody, Sebastian Zoellner, Charles Langelier, Prescott G. Woodruff, Tuuli Lappalainen, Stephanie A. Christenson

**Affiliations:** 1 New York Genome Center, New York, NY USA; 2 Department of Systems Biology, Columbia University, New York, NY USA; 3 Department of Internal Medicine, Section of Pulmonary, Critical Care, Allergy and Immunologic Diseases, Wake Forest School of Medicine, Winston-Salem, NC USA; 4 Department of Medicine, Stanford University School of Medicine, Stanford, CA USA; 5 Division of Pulmonary, Critical Care, Allergy, & Sleep Medicine, Department of Medicine, University of California San Francisco, San Francisco, CA USA; 6 Department of Medicine, Columbia University Medical Center, New York, NY USA; 7 Division of Pulmonary and Critical Care Medicine, Department of Medicine, David Geffen School of Medicine, University of California Los Angeles, Los Angeles, CA USA; 8 Division of Genetics, Genomics and Precision Medicine, Department of Medicine, University of Arizona, Tucson, AZ USA; 9 Division of Pulmonary Medicine, Department of Medicine, National Jewish Health, Denver, CO USA; 10 Division of Pulmonary and Critical Care, University of Iowa, Iowa City, IA USA; 11 Department of Biostatistics, University of North Carolina at Chapel Hill, Chapel Hill, NC USA; 12 Department of Thoracic Medicine and Surgery, Lewis Katz School of Medicine at Temple University, Philadelphia, PA USA; 13 Division of Pulmonary and Critical Care Medicine, Department of Medicine, University of Michigan Health System, Ann Arbor, MI USA; 14 Medicine Service, VA Ann Arbor Healthcare System, Ann Arbor, MI USA; 15 Division of Pulmonary and Critical Care Medicine, Department of Medicine, Johns Hopkins School of Medicine, Baltimore, MD USA; 16 Division of Physiologic Imaging, Department of Radiology, University of Iowa Hospitals and Clinics, Iowa City, IA USA; 17 Division of Pulmonary and Critical Care Medicine, Department of Internal Medicine, Weill Cornell Medicine, New York, NY USA; 18 Department of Genetic Medicine, Weill Cornell Medicine, New York, NY USA; 19 Division of Pulmonary, Critical Care, Sleep and Allergy, University of Illinois at Chicago, Chicago, IL USA; 20 Division of Pulmonary, Allergy and Critical Care Medicine, Department of Medicine, University of Alabama at Birmingham, Birmingham, AL USA; 21 Division of Pulmonary and Critical Care Medicine, Department of Internal Medicine, University of Utah, Salt Lake City, UT USA; 22 Division of Pulmonary, Critical Care and Sleep Medicine, Department of Internal Medicine, University of Kansas School of Medicine, Kansas City, KS USA; 23 Division of Allergy, Pulmonary, and Critical Care Medicine, Department of Medicine, University of Wisconsin-Madison, Madison, WI USA; 24 Department of Pathobiology, Lerner Research Institute, Cleveland Clinic, Cleveland, OH USA; 25 Division of Pulmonary and Critical Care Medicine, Department of Medicine, Brigham and Women’s Hospital, Boston, MA USA; 26 Department of Environmental and Occupational Health, Graduate School of Public Health, University of Pittsburgh, Pittsburgh, PA USA; 27 Respiratory Institute, Cleveland Clinic, Cleveland, OH USA; 28 Division of Infectious Diseases, University of California San Francisco, San Francisco, CA USA; 29 Chan Zuckerberg Biohub, San Francisco, CA USA

**Keywords:** COVID-19, SARS-CoV-2, ACE2, eQTL, Bronchial epithelium

## Abstract

**Background:**

The large airway epithelial barrier provides one of the first lines of defense against respiratory viruses, including SARS-CoV-2 that causes COVID-19. Substantial inter-individual variability in individual disease courses is hypothesized to be partially mediated by the differential regulation of the genes that interact with the SARS-CoV-2 virus or are involved in the subsequent host response. Here, we comprehensively investigated non-genetic and genetic factors influencing COVID-19-relevant bronchial epithelial gene expression.

**Methods:**

We analyzed RNA-sequencing data from bronchial epithelial brushings obtained from uninfected individuals. We related *ACE2* gene expression to host and environmental factors in the SPIROMICS cohort of smokers with and without chronic obstructive pulmonary disease (COPD) and replicated these associations in two asthma cohorts, SARP and MAST. To identify airway biology beyond *ACE2* binding that may contribute to increased susceptibility, we used gene set enrichment analyses to determine if gene expression changes indicative of a suppressed airway immune response observed early in SARS-CoV-2 infection are also observed in association with host factors. To identify host genetic variants affecting COVID-19 susceptibility in SPIROMICS, we performed expression quantitative trait (eQTL) mapping and investigated the phenotypic associations of the eQTL variants.

**Results:**

We found that *ACE2* expression was higher in relation to active smoking, obesity, and hypertension that are known risk factors of COVID-19 severity, while an association with interferon-related inflammation was driven by the truncated, non-binding *ACE2* isoform. We discovered that expression patterns of a suppressed airway immune response to early SARS-CoV-2 infection, compared to other viruses, are similar to patterns associated with obesity, hypertension, and cardiovascular disease, which may thus contribute to a COVID-19-susceptible airway environment. eQTL mapping identified regulatory variants for genes implicated in COVID-19, some of which had pheWAS evidence for their potential role in respiratory infections.

**Conclusions:**

These data provide evidence that clinically relevant variation in the expression of COVID-19-related genes is associated with host factors, environmental exposures, and likely host genetic variation.

**Supplementary Information:**

The online version contains supplementary material available at 10.1186/s13073-021-00866-2.

## Background

Coronavirus disease 2019 (COVID-19), the clinical syndrome caused by the severe acute respiratory syndrome coronavirus 2 (SARS-CoV-2) virus, has led to a global crisis. As a respiratory virus, SARS-CoV-2 is hypothesized to gain entry into humans via the airway epithelium, where it initiates a host response that leads to the subsequent clinical syndrome. Despite an immense global burden of disease, the manifestations of SARS-CoV-2 infection vary enormously, from asymptomatic infection to progressive acute respiratory failure and death. The viral or host features that determine the course of disease in each individual are poorly understood. Multiple clinical risk factors for severe COVID-19 have been identified, including older age, male sex, African American race, smoking, and comorbidities such as hypertension, obesity, diabetes, cardiovascular disease, and chronic airway diseases [1–5], as well as host genetics [5–8].

**Fig. 1:**
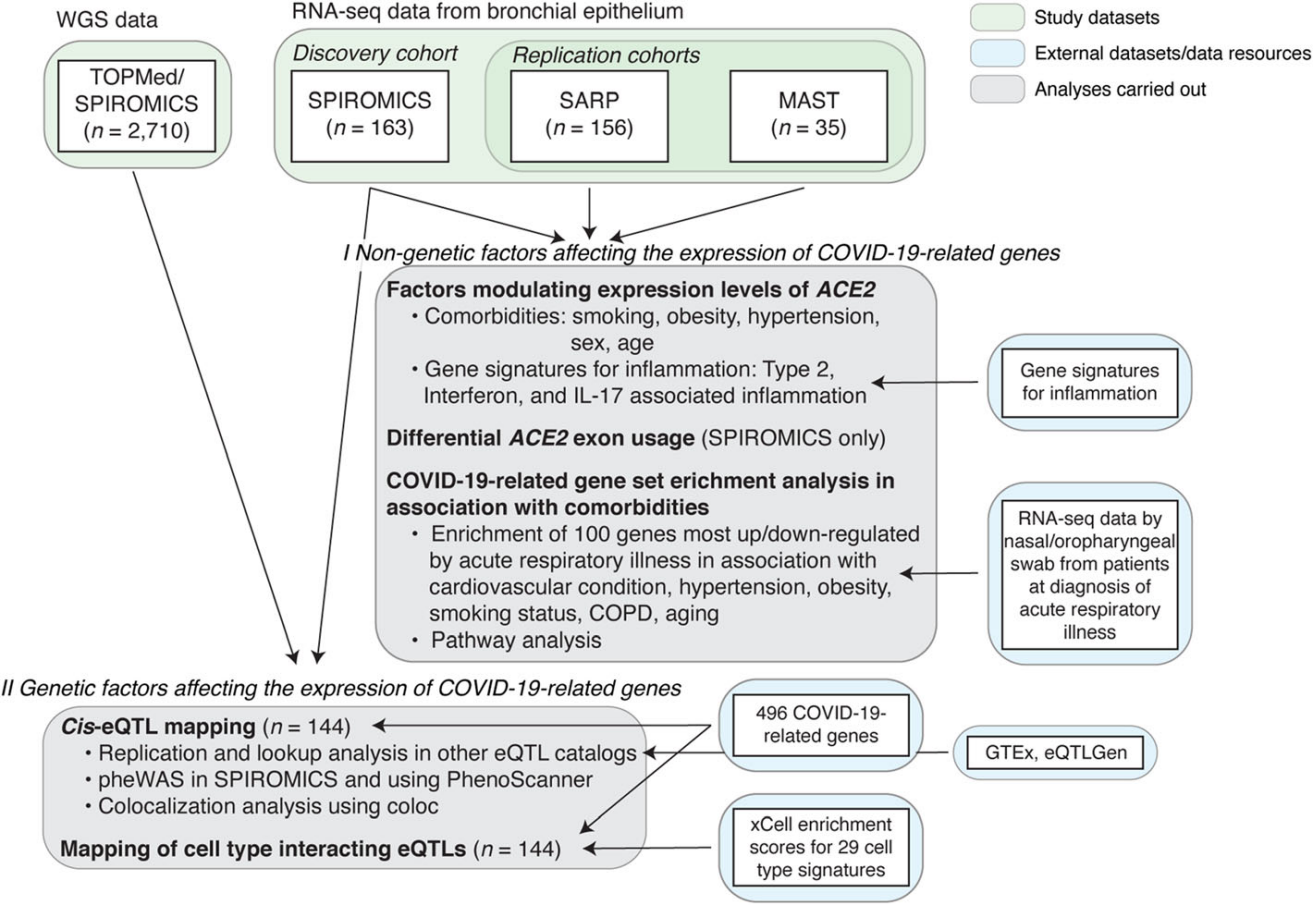
Study design. Graphical illustration of analyses (gray boxes) carried out to study non-genetic and genetic factors affecting the expression of COVID-19-related genes in bronchial epithelium. Input data sets for these analyses are denoted with a green box (WGS and RNA-seq) and external data sets or data resources used in these analyses are denoted with a blue box

The expression levels of genes that interact with the SARS-CoV-2 virus or are involved in the subsequent host response are hypothesized to be an important host factor that could partially underlie the substantial inter-individual variability in COVID-19 susceptibility and progression [9–11]. To this end, we investigate genetic and non-genetic factors influencing the expression of human genes that have been implicated in COVID-19 (study design in Fig. 1). We analyze RNA-sequencing (RNA-seq) data from bronchial brushing samples obtained from the SPIROMICS cohort (*n* = 163) [12], notable for the high burden of COVID-19-relevant comorbidities and rich phenotype and whole genome sequencing (WGS) data from the TOPMed Project [13]. For replication, we use two asthma RNA-seq data sets, SARP (*n* = 156) and MAST (*n* = 35) as well as expression quantitative trait loci (eQTL) data from GTEx [14]. Our analysis provides insights of the contribution of host factors and host genetics in the expression of COVID-19-related genes in the large airway epithelium for understanding inter-individual variation of COVID-19.

## Methods

### Study population

#### SubPopulations and InteRmediate Outcome Measures In COPD Study (SPIROMICS)

SPIROMICS is a multi-site prospective cohort study in which the main objective is to identify subpopulations of chronic obstructive pulmonary disease (COPD) as well as markers of disease severity to enable targeted treatment and disease modification. Data were obtained from participants who underwent research bronchoscopy within SPIROMICS between February 1, 2012, and May 31, 2016. Participants ages 40–80 were enrolled across four strata (never smokers, smokers without COPD, mild/moderate COPD, and severe COPD). Full SPIROMICS study details including inclusion and exclusion criteria have been previously published [12]. Participants enrolled in SPIROMICS who consented to a research bronchoscopy and met all local requirements (e.g., any laboratory tests that are required by institutional policy to be administered prior to a bronchoscopy) were deemed eligible. Additional exclusion criteria for the SPIROMICS bronchoscopy sub-study [15] included history of cardiac disease or other comorbid condition severe enough to significantly increase risks based on investigator discretion, requirement of supplementary oxygen at rest based on arterial oxygen pressure less than 60 mmHg or arterial oxygen saturation less than 88%, severe lung function impairment defined as post-bronchodilator forced expiratory volume in 1 s (FEV1) less than 30% predicted, and use of anticoagulation or antiplatelet therapies.

#### Severe Asthma Research Program (SARP)

SARP is a prospective multi-center cohort study with a primary goal of improving the mechanistic and clinical understanding of severe asthma [16]. Adult and pediatric patients with and without asthma were recruited to the SARP III cohort between November 1, 2012, and October 1, 2014, by seven clinical research centers in the USA. The SARP protocol is an ongoing, six-visit, 3-year, longitudinal cohort study in which 60% of participants have severe asthma as defined by the European Respiratory Society/American Thoracic Society (ERS/ATS) criteria [17]. A subset of participants underwent research bronchoscopy. Exclusion criteria included history of smoking (> 5 pack year smoking history), co-existing lung disease, and uncontrolled comorbidities. All healthy control subjects had to have no history of asthma and normal lung function and methacholine bronchoprovocation testing. Participants with asthma had to meet ERS/ATS criteria for asthma (bronchodilator response to albuterol or positive methacholine bronchoprovocation test). Asthma had to be clinically stable at the time of bronchoscopy.

#### Mechanisms of ASThma study (MAST)

MAST is a single-center clinical study with a primary objective of understanding asthma biology through detailed analyses of airway secretions and tissues [18]. Mild steroid-naive asthmatics and healthy controls underwent research bronchoscopy between April 2007 and December 2011. All healthy control subjects had to have no history of asthma or allergies. Participants with asthma had to have a positive methacholine bronchoprovocation test and could not have used steroids in 6 weeks prior to enrollment. Additional exclusion criteria included respiratory infection within 4 weeks of enrollment and pregnancy.

### Whole genome sequencing data

Trans-Omics for Precision Medicine (TOPMed) Project [13] data freeze 9 consist of whole genome sequences of 160,974 samples with at least 15x average coverage, including 2710 individuals from the SPIROMICS study. We obtained unphased genotypes for all individuals from the SPIROMICS study at sites with at least 10x sequencing depth (minDP10 call set) aligned to the human reference genome build GRCh38. Details regarding the DNA sample handling, quality control, library construction, clustering and sequencing, read processing, and sequence data quality control are described on the TOPMed website (https://www.nhlbiwgs.org/genetic). Variants passing all quality control (QC) filters were retained.

### Derivation of airway epithelial transcriptomic data in SPIROMICS, SARP, and MAST

Cytological brushings of the airway epithelium were obtained from lower lobe bronchi at the segmental or subsegmental carina. RNA was isolated with miRNeasy extraction kits (Qiagen Inc., Valencia, CA). RNA quantity and quality were evaluated using a NanoDrop Spectrophotometer (Thermo Fisher Scientific, Wilmington, DE) and Agilent 2100 Bioanalyzer (Agilent Technologies, Santa Clara, CA), respectively. Library preparation with multiplexing was performed using Illumina TruSeq Stranded Total RNA with Ribo-zero GOLD kit (SPIROMICS, SARP) or Human/Mouse/Rat kit (MAST) per manufacturer’s protocol. Samples were sequenced using one-hundred-fifty base-pair (SPIROMICS) or one-hundred base-pair (SARP, MAST) paired end reads via the Illumina HiSeq platform at the UCSF Sandler Genomics core. FASTQ files were quality filtered and aligned to the Ensembl GRCh38 genome build using STAR [19]. Read counts were normalized using the regularized logarithm transformation function of the DESeq2 package in R [20] and batch corrected using the Combat function in the SVA package in R [21]. Outlying samples with low quality (low raw read counts, high percentage of reads mapped to multiple loci, high percentage of unmapped reads) were identified by hierarchical clustering and principal component analyses and excluded from the final data sets.

### Differential expression analysis of *ACE2* in relation to host/environmental factors

Linear regression models were fitted to evaluate associations between *ACE2* expression (based on normalized count) and clinical variables in the SPIROMICS, SARP, and MAST cohorts with and without adjustments for covariates (see Additional file 1 for additional details).

### Differential exon usage

Following alignment, we indexed and sliced the SPIROMICS BAM files to include 51.6 kb of the *ACE2* genomic region (chrX:15,556,393-15,608,016 in the hg38 genome build) using samtools [22]. GTF files were manually curated to include the three exons that contribute to differential isoform expression of *ACE2* [23]. Of them, the truncated *ACE2* transcript (*dACE2*) that does not bind the SARS-CoV-2 virus but is associated with an interferon-stimulated gene response in experimental models originates from Exon 1c. The exons were counted using the ASpli package in R [24]. After correcting for overall gene counts and differences in sequence depth, linear models adjusting for batch were used to analyze differences in exon usage in association with interferon-stimulated gene signature and clinical covariates. Interpretation of differential exon usage requires consideration of the necessary adjustment for variation in total transcript count. Thus, if overall *ACE2* expression is decreased in association with an outcome, a differential increase in one exon adjusts the expression of that isoform away from the overall negative association, but does not necessarily mean that the isoform is not negatively associated with the outcome to a lesser extent. Further details are provided in Additional file 1.

### Gene set enrichment analysis of expression changes induced by COVID-19

We built COVID-19-relevant gene sets from publicly available differential gene expression data from participants who underwent nasal/oropharyngeal swab sampling at the time of acute respiratory illness for COVID-19 diagnosis (94 participants with COVID-19, 41 with other viral illness, 103 with no virus identified, viruses identified by metagenomic sequencing analysis) using Supplementary File 1 from Mick et al. [25]. Biological pathway gene sets were built by inputting the genes differentially downregulated between SARS-CoV-2 infection and other viral illness (*P* < 0.05) into the Ingenuity Pathway Analysis canonical pathway function. Gene set enrichment analyses were then performed using FGSEA [26] and the CAMERA function [27] in limma against gene lists ranked by their log fold change differential expression in association with comorbid clinical risk factors. Barcode plots were made using CAMERA. Findings were considered significant at *P* < 0.05 and false discovery rate (FDR) < 0.05 if multiple corrections were necessary. Additional details are provided in Additional file 1.

### COVID-19-related genes

We selected 514 candidate genes implicated in COVID-19 from six different sources: Hoffmann et al. [28], Gordon et al. [29], Blanco-Melo et al. [30], COVID-19 Cell Atlas (https://www.covid19cellatlas.org/), Gassen et al. [31], and Wang et al. [32]. Of them, 496 genes were expressed in bronchial epithelium in the SPIROMICS cohort. Further details are provided in Additional file 1.

### Expression quantitative trait mapping

Expression quantitative trait locus (eQTL) mapping was performed in 144 unrelated individuals from the SPIROMICS bronchoscopy sub-study with WGS genotype data from TOPMed and gene expression from bronchial epithelium profiled with RNA-seq following the analysis pipeline from the Genotype-Tissue Expression (GTEx) Consortium [14]. In short, gene expression data was normalized as follows: (1) read counts were normalized between samples using TMM [33] with edgeR [34], (2) genes with TPM ≥ 0.1 and unnormalized read count ≥6 in at least 20% of samples were retained, and (3) expression values were transformed using rank-based inverse normal transformation across samples.

*Cis*-eQTL mapping was performed using tensorQTL [35] across 22,738 genes and 6,605,907 variants with minor allele frequency (MAF) ≥ 0.05 and variant call rate ≥ 0.9 within ± 1 Mb from the transcription start site (TSS) of the gene. As covariates in the model, we used 15 PEER factors [36], 4 genotype principal components and sex imputed from genotype data. To control for multiple testing, 10,000 permutations were performed and FDR < 0.05 was used to identify genes with statistically significant eQTLs (eGenes). Lead *cis*-eQTL effect size was quantified as allelic fold change (aFC) [37], ratio of expression of the haplotype carrying the alternative allele to expression of the haplotype carrying the reference allele of an eQTL.

Additionally, cell type interacting eQTLs (ieQTLs) were mapped using an interaction model: *p* ~ *g* + *i* + *g* × *i* + *C*, where *p* is the expression vector (normalized as described above), *g* is the genotype vector, *i* is the normalized cell type enrichment score from xCell [38], *g × i* is the interaction term, and *C* is the covariates matrix as used in standard eQTL mapping. Only variants with MAF > 0.1 in the samples belonging to the top and bottom halves of the distribution of cell type abundance were included in the analyses. Multiple testing correction was done at the gene level using eigenMT [39], followed by Benjamini-Hochberg procedure across genes at FDR 5%. Additional details are provided in Additional file 1.

### Replication of *cis*-eQTLs and pathway analysis

We performed replication of *cis*-eQTLs (gene-variant pairs) found from bronchial epithelium in 49 tissues from the GTEx project v8 release [14] based on the proportion of true positives [40], π_1_, and concordance rate, the proportion of gene-variant pairs with the same allelic direction for variants with nominal *P* value < 1 × 10^−4^ in the given GTEx tissue. Then, we analyzed the replication and concordance measure as a function of sample size and median cell type enrichment scores for seven cell types [41]. Further details are provided in Additional file 1.

Also, we performed gene-level lookup in GTEx v8 and eQTLGen Consortium [42] and used the functional profiling webtool g:GOSt from g:Profiler [43] to perform pathway analysis of the 492 significant eGenes in SPIROMICS not tested in GTEx v8 Lung.

### pheWAS of lead COVID-19 *cis*-eQTLs in SPIROMICS and querying PhenoScanner

We performed a phenome-wide association study (pheWAS) in 1980 non-Hispanic White and 696 individuals from other ethnic and racial groups from SPIROMICS for the 108 lead *cis*-eQTLs to evaluate for phenotypic associations with spirometric measures, cell count differentials, and other variables. PheWAS regression-based models were performed using PLINK 2/0 adjusting for principal components of ancestry, sex, body mass index (BMI), age, and smoking pack-years. Significance threshold was set for the number of eQTLs tested across phenotypes (*P* < 4.63 × 10^−4^).

Additionally, PhenoScanner v2 [44, 45] was used to lookup phenotype associations of the *cis*-eQTL variants from large-scale genome-wide association studies (GWAS) with association *P* value < 10^−5^. The phenoscanner R package (https://github.com/phenoscanner/phenoscanner) was used to perform the queries. Additional details are provided in Additional file 1.

### Colocalization analysis

To assess evidence for shared causal variant of a *cis*-eQTL and a GWAS trait, we used the Bayesian statistical test for colocalization, coloc [46], with conditioning and masking to overcome one single causal variant assumption. Coloc was run on a 500-kb region centered on the lead *cis*-eQTL with priors set to *p*_*1*_ = 10^−4^, *p*_*2*_ = 10^−4^, *p*_*3*_ = 5 × 10^−6^. We used the coloc.signals() function with mode = iterative, method = mask for GWAS traits with linkage disequilibrium (LD) data from the 1000 Genomes Project, and method = single for the eQTLs. Posterior probability for colocalization (PP4) > 0.5 was used as evidence for colocalization (see Additional file 1 for further details).

## Results

### Smoking, obesity, and hypertension are associated with increased airway epithelial expression of functional ACE2 isoforms

**Fig. 2:**
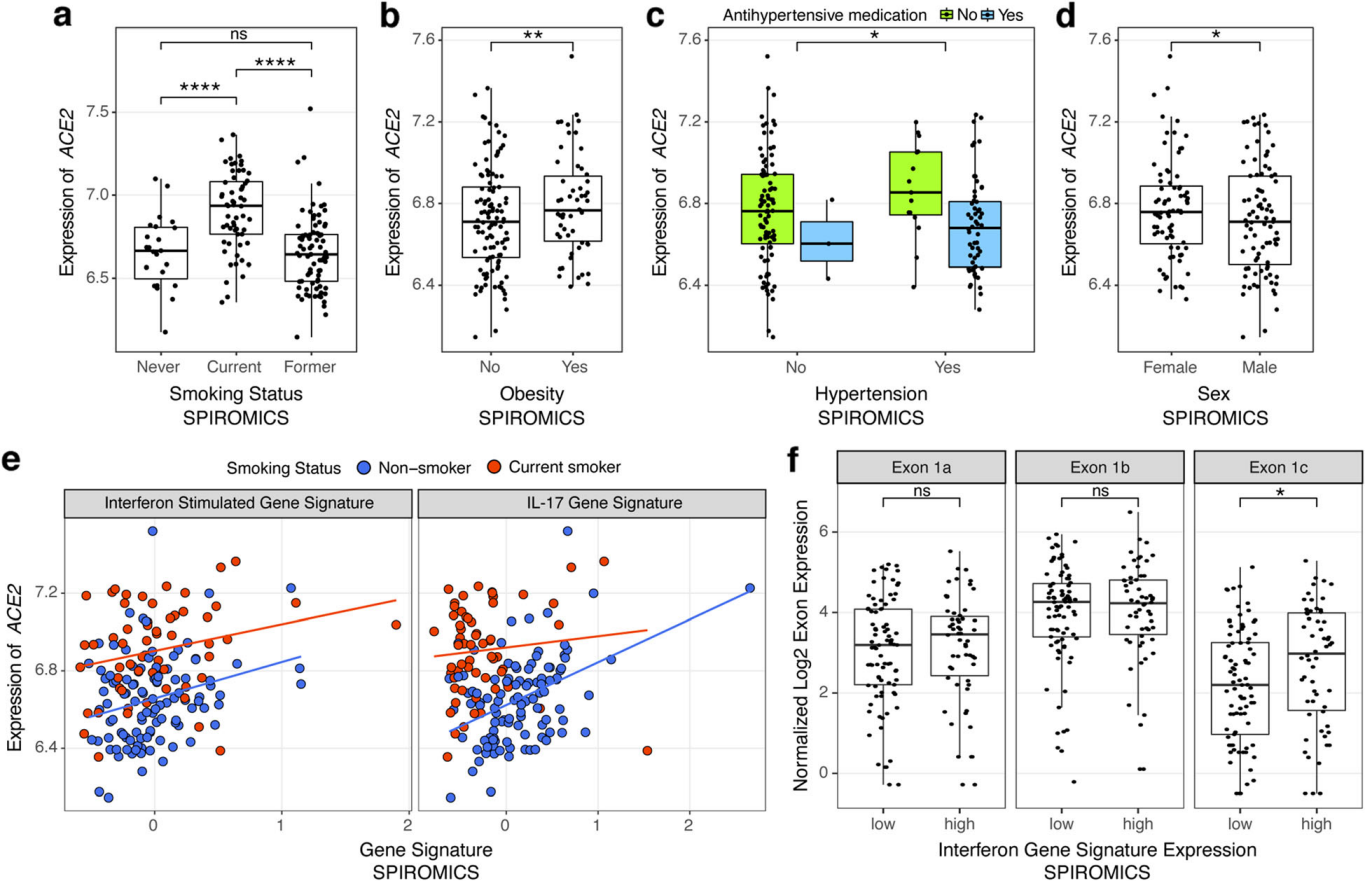
*ACE2* gene expression associations in SPIROMICS. **a**–**d** Box plots showing that *ACE2* log_2_ gene expression (*x*-axis) was increased in association with current but not former smoking as compared to never smokers (**a**), obesity (**b**, validated in the MAST and SARP cohorts, Additional file 3: Figure S2a-b), hypertension (**c**, adjustments include anti-hypertensive treatment, validated in SARP, Additional file 3: Figure S3a, data not collected in MAST), and female sex (**d**, not replicated in either MAST or SARP, Additional file 2: Table S1A). **e** Scatterplots showing that *ACE2* gene expression was increased in association with higher levels of our previously validated gene signatures of the airway epithelial response to interferon (left panel, replicated in SARP) and to IL-17 inflammation (right panel, replicated in MAST and SARP) after adjusting for smoking status (Additional file 2: Table S1B). **f** Box plots showing that *ACE2* Exon 1c, which contributes to the truncated *ACE2* transcript was differentially increased in association with our interferon signature while Exons 1a and 1b that contribute to the full length *ACE2* transcript were not. *P* values indicated by: **** < 0.0001, *** < 0.001, ** < 0.01, * < 0.05, ns = not significant in linear models adjusted for covariates. In **a**–**d** and **f**, the boxes denote the interquartile range, the center line denotes the median, and whiskers denote the interquartile range × 1.5

We first analyzed expression levels of *ACE2*, the receptor of the SARS-CoV-2 Spike protein that is the key host gene for viral entry [28, 47], in relation to non-genetic host factors (Additional file 2: Table S1). Corroborating previous reports [11, 48–50], we found that current smoking, when compared to non-smoking, had the largest overall effect on *ACE2* expression of any phenotypic feature studied in SPIROMICS, before and after adjustments for covariates (log_2_ fold change (FC) = 0.30 ± 0.06, *P* = 1.7 × 10^−7^, Fig. 2a). This effect was absent in former smokers. In similarly adjusted models, we found no association between *ACE2* levels and COPD (Additional file 3: Figure S1a), nor with asthma in MAST [50] (Additional file 3: Figure S1c). In SARP, *ACE2* levels were slightly lower in asthmatics compared to healthy controls (Additional file 3: Figure S1b), which was largely driven by decreased expression of *ACE2* only in asthmatics on oral steroids (Additional file 3: Figure S1d). African American race was associated with increased *ACE2* expression in both SPIROMICS and SARP, but no association after adjusting for covariates suggests that this was due to a higher prevalence of comorbid conditions (Additional file 3: Figure S1e-f). However, *ACE2* expression was significantly higher across data sets in association with two relevant comorbidities, obesity and hypertension (Fig. 2b-c, Additional file 3: Figure S2a-e, Additional file 3: Figure S3a-b). Of note, we further found that use of anti-hypertensives in SPIROMICS attenuates the association between *ACE2* and hypertension towards levels seen in non-hypertensive participants (Fig. 2c). When stratified by anti-hypertensive class, angiotensin receptor blockers (ARBs) and diuretics, but not ACE inhibitors or calcium channel blockers, were associated with lower *ACE2* levels, partially dependent on smoking status (Additional file 3: Figure S3c). Counterintuitively, modest decreases in *ACE2* expression were seen in SPIROMICS in association with age (log_2_ FC = − 0.064 ± 0.02, *P* = 0.005 for every 10-year age increase, Additional file 3: Figure S4a) and male sex (log_2_ FC = − 0.076 ± 0.035, *P* = 0.03, Fig. 2d) before and after adjustments, although similar associations were not seen in SARP or MAST. Although there were no significant differences in the above reported outcomes between males and females in SPIROMICS, former smokers were older (9.1 ± 1.3 years compared to current smokers, *P =* 3.19 × 10^−10^) as were participants with hypertension (4.6 ± 1.4 years, *P =* 0.002, Additional file 3: Figure S5). Sex and age were, however, both adjusted for in our analyses.

As chronic airway inflammation, prevalent but heterogeneous in the airway diseases studied in the included cohorts, can influence gene expression and the host response to infections, we next studied how stereotypic adaptive airway immune responses affect *ACE2* expression. We used our previously validated gene expression signatures to quantify type 2-, interferon-, and IL-17-associated inflammation [18, 51, 52]. We found that *ACE2* expression was associated with increased interferon-related inflammation, as previously reported [9], as well as IL-17-related but not type 2 inflammation across data sets (Fig. 2e). Corroborating the association with IL-17 inflammation, genes highly co-expressed with *ACE2* expression included genes in our IL-17 signature across data sets (Additional file 2: Table S2).

Recent reports suggested that *ACE2* induction by interferon stimulation may be explained by expression of a truncated *ACE2* isoform (*dACE2*, initiated from exon 1c instead of 1a/b) that does not bind the SARS-CoV-2 spike protein [23, 53]. We first corroborated this finding, showing that our interferon-stimulated gene signature is associated with increased exon 1c but not exons 1a or 1b usage (Fig. 2f). We also identified an increase in exon 1a usage with age. This result suggests that although overall *ACE2* expression is decreased in association with age, the full length transcript initiated from exon 1a is not decreased to the same extent or is even potentially increased with age (Additional file 3: Figure S4b). Importantly, differential exon 1c usage was not associated with any other clinical/biological outcomes of interest, suggesting that the full length transcript is responsible for the observed associations.

These results overall indicate that smoking, obesity, and hypertension affect airway epithelial expression of functional *ACE2* isoforms, as previously shown for smoking [11, 48–50]. The *ACE2* association with interferon-related inflammation appears to be explained by the truncated version of *ACE2* [23, 53]. Together, these findings suggest that smoking, obesity, and hypertension may contribute to COVID-19 severity through an association with increased *ACE2* expression, while other risk factors such as male sex and airway disease likely contribute via other mechanisms, corroborating recent evidence on sex differences in the immune response to COVID-19 [54].

### Obesity, hypertension, and cardiovascular disease are associated with a relative COVID-19-relevant immunosuppression at the airway epithelium

As the host’s ability to mount an appropriate response to respiratory viruses may alter susceptibility to severe infection, we next performed gene set enrichment analyses (GSEA) to determine whether clinical risk factors are associated with similar airway gene expression patterns indicative of a diminished immune response that we recently identified early in COVID-19 by nasal/oropharyngeal swab [25]. As we previously reported, the genes differentially expressed in association with SARS-CoV-2 infection compared to other viruses at diagnosis indicate a diminished innate and adaptive immune response that may allow for unabated viral infection and account for the long pre-symptomatic period associated with COVID-19 [25]. We hypothesized that clinical risk factors uniquely associated with COVID-19 severity (e.g., cardiovascular disease, hypertension) could predispose patients to develop more severe disease by contributing to this relative immunosuppression. We derived gene sets from our previously published RNA-seq data collected by nasal/oropharyngeal swab from patients at diagnosis of acute respiratory illness; 94 had COVID-19, 41 had other viral illness, and 103 had no virus identified by metagenomic sequencing analysis [25]. First, we generated gene sets derived from the 100 genes most up- and downregulated in association with infection type to use to determine if there were global similarities in gene expression changes across data sets. For pathway analyses, we then generated COVID-19-relevant gene sets specific to particular canonical pathways by inputting significantly differentially expressed genes (*FDR* < 0.05) between SARS-CoV-2 infection and other viral respiratory illness into the Ingenuity Pathway Analysis (IPA) canonical pathway function (Additional file 2: Table S3). GSEA was then performed using FGSEA [26] in which these gene sets were tested against gene lists ranked by their log fold change differential expression in association with comorbid clinical risk factors.

**Fig. 3:**
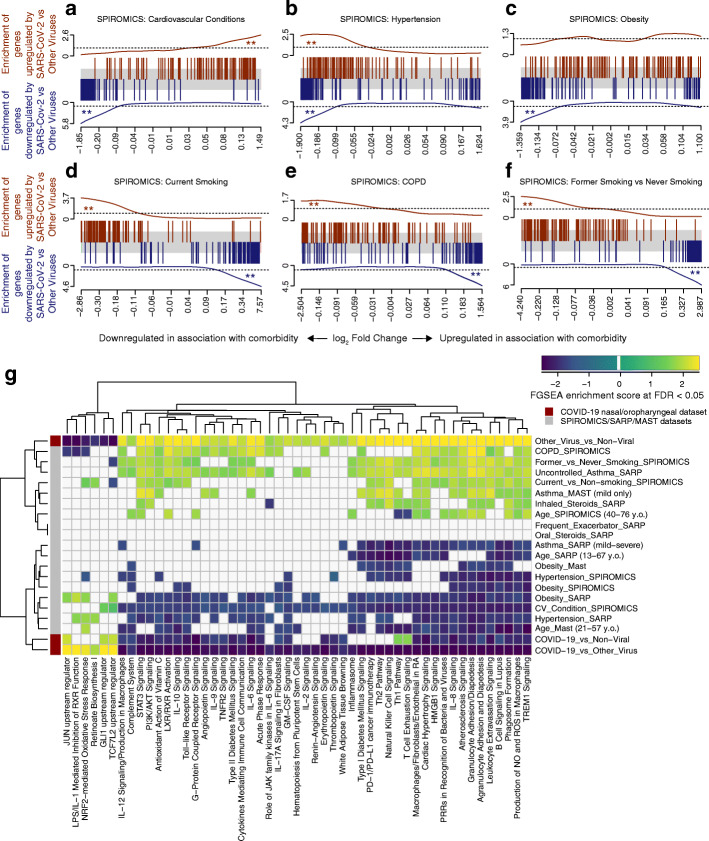
COVID-19-related gene set enrichment analyses in association with comorbidities. **a**–**f** Barcode plots in which the vertical lines represent the 100 genes most upregulated (red) or downregulated (blue) in nasal/oropharyngeal swab samples obtained from COVID-19 patients as compared to other viruses at the time of diagnosis of an acute upper respiratory infection. These gene sets are plotted against log fold gene expression changes arranged from most downregulated to most upregulated with that comorbidity (horizontal gray bar). Lines above (red) and below (blue) the bar represent the running sum statistic with a significant finding indicated when the line crosses the dashed line at either end of the plot. Genes downregulated by SARS-CoV-2 infection compared to other viruses were significantly enriched amongst genes downregulated in association with cardiovascular conditions overall (**a**), hypertension (**b**), and obesity (**c**), while in current (**d**) and former smoking (**f**) and in COPD (**e**), these downregulated genes in COVID-19 were enriched amongst upregulated genes in association with comorbidity. ** indicates FDR < 0.05. **g** COVID-19-related pathway gene sets were generated from an IPA analysis of the genes downregulated by SARS-CoV-2 infection compared to other viruses. Gene set enrichment scores for gene sets enriched at FDR < 0.05 (columns) are shown in the heatmap plotted against comorbidities (rows) with gene sets enriched amongst downregulated and upregulated genes indicated in blue and yellow, respectively. All pathways not enriched at FDR < 0.05 were shrunk to zero (white). Euclidean distance with average linkage was used for clustering

We found that the genes most downregulated in association with SARS-CoV-2 infection as compared to other viruses were significantly enriched amongst genes downregulated in association with obesity, hypertension, and cardiovascular disease in SPIROMICS (Fig. 3a–c). Findings for obesity were replicated in SARP and MAST and for hypertension in SARP (Additional file 3: Figure S6a-c, hypertension data not collected in MAST, cardiovascular disease data not collected in SARP or MAST). Conversely, genes upregulated in other viral infections (or conversely, downregulated by SARS-CoV-2) were upregulated in inflammatory airway conditions (current and former smokers, COPD) (Fig. 3d-f). Aging was associated with an enrichment in genes downregulated by SARS-CoV-2 infection only in MAST while genes upregulated with SARS-CoV-2 infection were enriched with increasing age across the data sets (Additional file 3: Figure S6d-f). Our results demonstrate a sharp contrast between SARS-CoV-2 and other viral infections, which often trigger airway disease exacerbations by potentiating the chronic airway inflammation associated with these diseases and smoking exposure. We found this same pattern in association with asthma in MAST but not when considering asthma overall in SARP, potentially due to heterogeneity of its asthma subjects. When considering just asthmatics with uncontrolled symptoms or those on inhaled compared to no steroids (a marker of severity), we did find this same enrichment of genes up and downregulated in association with non-COVID viral infections (pathway enrichment shown in Fig. 3g).

We used pathway gene set enrichment to determine the potential biological significance of these findings. We found across data sets that pathway gene sets derived from genes downregulated by SARS-CoV-2 infection as compared to other viruses were also enriched amongst genes downregulated in association with obesity, hypertension, cardiovascular disease, and aging (FDR < 0.05, Fig. 3g, Additional file 2: Table S4). Enriched downregulated pathways included those related to pro-inflammatory cytokines such as IL-6 and IL-17 as well as macrophage and granulocyte activation. Furthermore, pathways related to cardiovascular and metabolic disease signaling such as atherosclerosis and diabetes signaling were also enriched. We confirmed the enriched findings by separately performing IPA canonical pathway analyses on the genes differentially expressed (*P* < 0.05) in association with these comorbidities, finding similar results in these global/unsupervised analyses (Additional file 2: Table S5). Conversely, pro-inflammatory airway conditions such as smoking and COPD led to opposite effects. These findings suggest that obesity, hypertension, cardiovascular disease, and age are associated with a relative COVID-19-relevant immunosuppression at the airway epithelium, which, by stunting early anti-viral host responses, could contribute to increased susceptibility to SARS-CoV-2 infection and disease severity.

### Host genetics has a biologically meaningful effect on the airway epithelial expression of many COVID-19-related genes

**Fig. 4:**
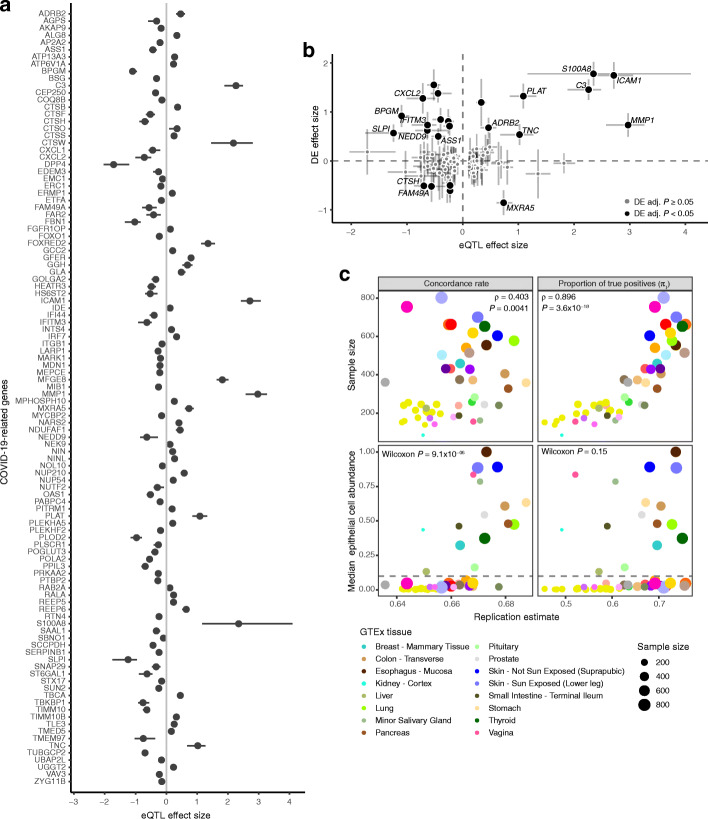
*Cis*-eQTLs in bronchial epithelium. **a** Effect size measured as allelic fold change (aFC, log_2_) of the significant *cis*-eQTLs for COVID-19 candidate genes. Error bars denote 95% bootstrap confidence intervals. **b** Comparison of the regulatory effects and the effect of SARS-CoV-2 infection on the transcription of COVID-19 candidate genes in normal bronchial epithelial cells from Blanco-Melo et al. [30]. The graph shows regulatory effects as aFC as in **a** and fold change (log_2_) of differential expression comparing the infected with mock-treated cells with error bars denoting the 95% confidence interval. Genes with adjusted *P* value < 0.05 in the differential expression analysis are colored in black, genes with non-significant effect are colored in gray. Highlighted genes have eQTL effect size greater than 50% of the differential expression effect size on the absolute scale. DE—differential expression. **c** Replication of *cis*-eQTLs from bronchial epithelium in GTEx v8 using the concordance rate (proportion of gene-variant pairs with the same direction of the effect, left panel) and proportion of true positives (π_1_, right panel). Upper panel shows the effect of sample size on the replication and concordance measures quantified as Spearman correlation coefficient (ρ). Lower panel shows the replication and concordance measures as the function of epithelial cell enrichment of the tissues measured as median epithelial cell enrichment score from xCell. Gray dashed line denotes median enrichment score > 0.1, which classifies tissues as enriched for epithelial cells. Wilcoxon rank sum test was used to estimate the difference in replication estimates between tissues enriched or not enriched for epithelial cells. The 16 tissues enriched for epithelial cells are outlined in the figure legend, for the full legend see Additional file 3: Figure S9a

In order to map host genetic variants, we focused on 496 genes implicated in SARS-CoV-2 infection (Additional file 2: Table S6): *ACE2* and *TMPRSS2*, key genes for viral entry [28]; *CTSL, CTSB,* and *BSG*, which may have a role as alternative routes for viral entry [28, 32]; host genes with protein-protein interactions with viral proteins [29]; differentially expressed genes as a response to the infection in cultured airway epithelial cells [30]; genes involved in autophagy that might counteract viral infection [31]; and other high interest genes from the COVID-19 Cell Atlas. Our *cis*-eQTL mapping in SPIROMICS (*n* = 144) identified significant (genome-wide FDR < 0.05) genetic regulatory variation for 108 (21.8%) of these COVID-19-related genes (Fig. 4a, Additional file 2: Table S7), with many genes also having significant eQTLs in other tissues in GTEx [14] (Additional file 2: Table S8).

Given the sample size, we have good power to discover the vast majority of eQTLs with > 2-fold effect on gene expression [14]. Many of the genes have a substantial genetic effect on gene expression: for example, the MERS receptor *DPP4* [55] has a *cis*-regulatory variant rs6727102 where the alternative allele decreases expression by 3.3-fold (Fig. 4a). In 16 genes, the genetic regulatory effects were > 50% of the magnitude of the differential expression induced by SARS-CoV-2 infection [30] (Fig. 4b). While the key genes *ACE2* or *TMPRSS2* did not have eQTLs in bronchial epithelium (Additional file 3: Figure S7a-b), as previously reported [50], *TMPRSS2* has an eQTL in GTEx lung tissue. This is consistent with the lack of phenome-wide association signals [56] or COVID-19 GWAS association at these loci (round 3 meta-analyses by COVID-19 Host Genetics Initiative [8]), suggesting that genetic regulation of these two genes is unlikely to contribute to potential host genetic effects on COVID-19. Many of the genes analyzed for eQTLs had variation in expression associated to clinical factors and comorbidities, with current smoking associated with the highest number of up-and downregulated genes in association with comorbidity (Additional file 3: Figure S8a-b). Compared to *ACE2*, the effect of current smoking on the expression of *TMPRSS2* was modest (Additional file 3: Figure S7c), and as previously reported [10], expression levels of *TMPRSS2* were higher in asthmatic than healthy controls, but not in COPD, and it decreased in association with steroid use (Additional file 3: Figure S7d).

*Cis*-eQTLs from bronchial epithelium replicated at a high rate in those tissues from the GTEx v8 data set [14] that have a large sample size or high epithelial cell abundance (Fig. 4c, Additional file 3: Figure S9a-b), reflecting similarity in cell type composition manifesting in similarity of regulatory variant activity [14]. However, relative to GTEx lung, our bronchial epithelium eQTLs included genes enriched for sensory perception of chemical stimulus and smell (Additional file 2: Table S9). In total, 143 genes with eQTLs in SPIROMICS were not tested in GTEx nor eQTLGen Consortium [42], since bronchial epithelium is not well represented in previous eQTL catalogs. In addition to standard *cis*-eQTL mapping, we mapped cell type interacting eQTLs [41] but none were discovered for the COVID-19-related genes.

### Regulatory variants for COVID-19-related genes as host risk factors for COVID-19 susceptibility

To study the role of these regulatory variants in COVID-19 risk, we first analyzed eQTLs in the chromosome 3 locus with a significant association with hospitalization due to COVID-19 [8] (meta-analyses round 3) and severe COVID-19 with respiratory failure [5, 7]. We found no significant eQTLs in the bronchial epithelium for any of the six genes in this locus (Additional file 3: Figure S10a), suggesting that this genetic association may be driven by other tissues or cell types with a role in COVID-19. Moreover, these genes were rather lowly expressed in bronchial epithelium (Additional file 3: Figure S10b).

**Fig. 5:**
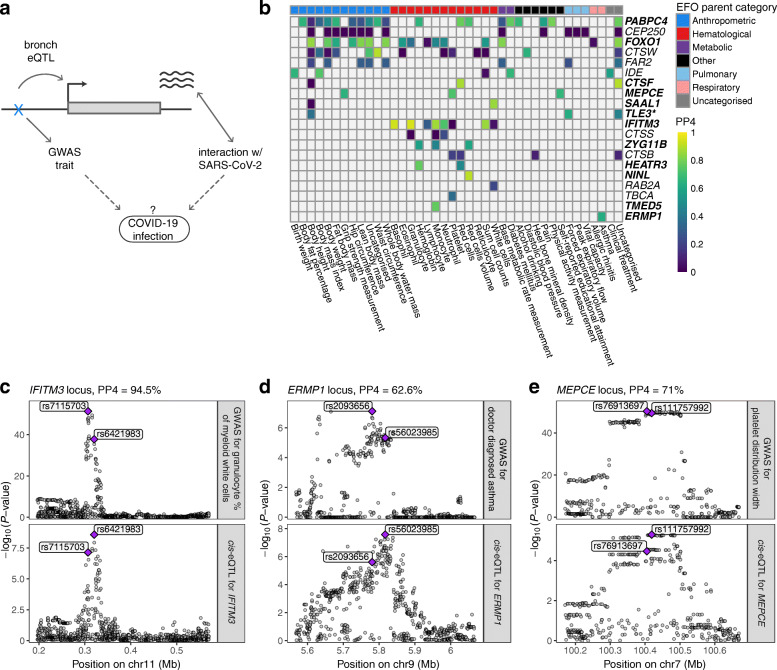
Colocalization analysis of the regulatory variants for COVID-19-related genes. **a** Illustration of the concept of how regulatory variants for COVID-19-related genes in bronchial epithelium can be possible candidates for genetic factors that affect infection or progression of the disease. Dotted lines denote the hypothesis we are able to create by searching for the phenotypic associations of the *cis*-eQTLs for COVID-19-related genes. **b** Heatmap of the colocalization analysis results for 20 COVID-19-related genes with eQTLs that have at least one phenotypic association belonging to the experimental factor ontology (EFO) parent categories relevant to COVID-19 (respiratory disease, hematological or pulmonary function measurement). Genes highlighted in bold indicate the loci involving COVID-19-relevant EFO categories with posterior probability for colocalization (PP4) > 0.5, suggesting evidence for shared genetic causality between eQTL and GWAS trait. In the *TLE* locus, the nearest genome-wide significant variant for forced expiratory volume in 1 s (FEV1) from Shrine et al. [57] is more than 1 Mb away, indicating that the association between the variant and FEV1 might be confounded by incomplete adjustment for height. **c**–**e** Regional association plot for the GWAS signal on the upper panel and *cis*-eQTL signal on the lower panel for *IFITM3* (**c**), *ERMP*1 (**d**), and *MEPCE* (**e**) locus, where the eQTL for the corresponding gene colocalizes with the GWAS trait relevant to COVID-19. Genomic position of the variants is shown on the *x*-axis and -log10(*P* value) of the GWAS or eQTL association on the *y*-axis. The lead GWAS and eQTL variants are highlighted

Next, given that COVID-19 GWAS still have limited power, we analyzed how regulatory variants for COVID-19-relevant genes associate to other immune- or respiratory-related phenotypes in large GWAS. Indication of these variants affecting (respiratory) infections would provide hypotheses of variants that might play a role in COVID-19 risk and its comorbidities (Fig. 5a). Thus, we performed a pheWAS analysis by Phenoscanner v2 [44, 45] for the 108 lead *cis*-eQTLs for COVID19-related genes and diverse set of phenotypes (Additional file 2: Table S10). Furthermore, we used the SPIROMICS phenotype data to study associations for 20 phenotypes (Additional file 2: Table S11). Of these loci, 44 were associated with at least one phenotype (*P* < 10^−5^), with expected patterns—best powered GWAS traits having most associations and shared signals for highly correlated traits (Additional file 3: Figure S11).

We further used colocalization analysis to extract loci where the eQTL and GWAS signals are likely to share a causal variant, as opposed to spurious overlap, focusing on 20 loci with associations for hematological and respiratory system traits of which 12 colocalized (PP4 > 0.5, Fig. 5b, Additional file 2: Table S12). In Fig. 5c, we highlight *IFITM3* that is upregulated by SARS-CoV-2 infection [30] and has an eQTL associated with multiple blood cell traits of the immune system [58] and neutrophil count in SPIROMICS (*P* < 0.002). In addition, *IFITM3* has a well-characterized role in the entry of multiple viruses, including coronaviruses [59]. Another interesting gene, *ERMP1* (Fig. 5d), has an eQTL colocalizing with an asthma GWAS association in the UK Biobank. ERMP1 interacts with the SARS-CoV-2 protein Orf9c [29] and ranks highly in a genome-wide CRISPR screen for genes required for SARS-CoV-2 infection [60]. Also, severe asthma is a risk factor for COVID-19 hospitalization [5] and death [61]. An eQTL for the *MEPCE* gene that interacts with SARS-Cov-2 protein Nsp8 [29] is associated with platelet parameters [58] (Fig. 5e). Interestingly, platelets are hyperactivated in COVID-19 [62, 63], and platelet count could be used as a prognostic biomarker in COVID-19 patients [64–66].

## Discussion

Using whole genome profiling data available from biologically relevant data sets, we have generated an archive of gene expression alterations that may contribute to COVID-19 susceptibility and severity. Although we include an extensive analysis of *ACE2* gene expression in bronchial epithelium and isoform usage, our findings extend beyond this, providing insight into the contribution of genetics and specific clinical risk factors in the airways’ response to the SARS-CoV-2 virus.

We demonstrate replicable associations between current smoking, obesity, hypertension, and increased bronchial epithelial *ACE2* expression, potentially facilitating SARS-CoV-2 entry into host cells. Obesity and hypertension have been strongly linked with COVID-19 susceptibility and severity [1–5]. We were not well-powered to study diabetes, but in a sputum gene expression study, we did find an association between diabetes and increased *ACE2* expression [67]. Together, this work suggests that one mechanism by which diseases associated with the metabolic syndrome are uniquely susceptible to COVID-19 is through increased *ACE2* expression. Although *ACE2* interacts with angiotensin 2 [68], we did not find that renin-angiotensin system-modifying drugs increased *ACE2* expression. In fact, although our sample size was small, our data suggests that angiotensin receptor blockers are associated with lower *ACE2* expression levels in smokers.

Although we observed that the largest increases in *ACE2* expression were amongst current smokers, active smoking has not been identified as one of the largest risk factors for COVID-19 [1–5]. Early reports suggested a lower prevalence of smoking amongst patients with COVID-19 as compared to the general population. However, these reports have been debunked as confounded and inappropriately designed based on the flawed assumption that individuals with symptomatic COVID-19 reflect the general population when they are actually older with more comorbidities [69]. Well-adjusted studies in COVID-19 have shown that current smoking is indeed associated with increased disease severity [70, 71]. Nonetheless, current smoking does not appear to be the biggest risk factor for developing severe COVID-19 disease in large clinical studies, and thus mechanisms beyond ACE2 receptor binding of the virus must be explored.

We find evidence that the truncated *dACE2* transcript is present in the bronchial epithelium and correlated with the expression of known interferon stimulated genes (ISGs). However, it does not appear to account for the observed clinical associations with overall *ACE2* expression. The functional role for *dACE2* is not currently known although it does not appear to bind SARS-CoV-2 [23, 53]. However, others have speculated [23] that during viral infections when ISGs are stimulated, *dACE2* may act as a dummy receptor for other *ACE2* ligands (e.g., microRNA-200c-3p) that if bound to *ACE2* would lead to internalization of the *ACE2*-ligand complex and functional depletion of *ACE2*. Thus, *dACE2* may keep *ACE2* levels high during infection. Our observations suggest that it is, however, the full length transcript and not this truncated isoform that is associated with clinical risk factors.

It is likely that much of the inter-individual variation in COVID-19 is driven by a more complex molecular response to the virus in the airway than expression of *ACE2* alone. This supposition is supported by our results demonstrating that obesity, hypertension, and cardiovascular comorbidities, as well as aging, are associated with a downregulation of mucosal immune response pathways similar to that seen in early SARS-CoV-2 infection in comparison to other viral infections. Together with clinical data and Mendelian randomization analyses of the causal role of smoking and BMI on severe COVID-19 [72], our result suggest that these important comorbidities increase COVID-19 susceptibility and severity by creating an airway microenvironment in which SARS-CoV-2 can gain a foothold before an effective host response is mounted. In SARS-CoV, a delayed innate immune response in tandem with early robust viral replication has been shown to lead to an enhanced late pro-inflammatory state and more severe lung injury [73]. Recent evidence suggests that SARS-CoV-2 may also impair early innate immune defenses through a host shutdown process [74]. Although diseases of the metabolic syndrome (e.g., cardiovascular conditions, obesity, and diabetes) are often associated with increased systemic inflammation, there is evidence of an associated delay in inflammatory cell recruitment to the lung during coronavirus infection in animal models [75, 76]. Further study of the lung-specific immune environment associated with these systemic diseases may be crucial to understanding susceptibility to severe SARS-CoV-2 infection.

In contrast to metabolic disorders, we find that inflammatory airway conditions increase gene expression indicative of increased innate and adaptive immune responses, potentially priming individuals for airway disease exacerbations in response to other viruses but not SARS-CoV-2. This is consistent with the large body of research showing that viruses trigger the majority of airway disease exacerbations [77]. SARS-CoV-2, however, appears to have a different immune profile and does not appear to be a major trigger for airway disease exacerbations in clinical studies [78, 79].

Furthermore, we show that host genetics has a biologically meaningful effect on the expression of many genes in the bronchial epithelium that may play an important role in COVID-19, including genes of interest as future drug targets that may not be covered by previous large eQTL catalogs from other tissue types. While we did not observe significant genetic regulatory effects for *ACE2* and *TMPRSS2*, the effect of regulatory variants on the expression of some COVID-19-related genes can be as strong as the expression changes induced by SARS-CoV-2 infection, highlighting the possible important role of host genetics in COVID-19. Most notably, 3p21.31 locus is robustly shown to be associated with COVID-19 severity [5, 7, 8], but the functional mechanisms are unclear. The six candidate genes—*SLC6A20*, *LZTFL1*, *CCR9*, *FYCO1*, *CXCR6*, and *XCR1*—were not highly expressed in bronchial epithelium, except for *LZTFL1*, and did not have eQTLs in our data set, suggesting that eQTL studies from other tissues and cell types could provide more evidence for the causative gene(s) of this genetic association.

Most severe cases of SARS-CoV-2 infection progress to acute respiratory distress syndrome and respiratory failure, thus regulatory variants for COVID-19-related genes that also affect respiratory infections or immune-related outcomes of a possible host response to a virus serve as candidates for host genetic factors for COVID-19, or its severity. We pinpoint multiple COVID-19-interacting genes for which genetic regulatory variants associate with immune- or respiratory-related outcomes, including the interferon-induced transmembrane protein 3 (*IFITM3*), endoplasmic reticulum metallopeptidase 1 (*ERMP1*), and methylphosphate capping enzyme (*MEPCE*), making them strong candidates for host genetic risk factors.

## Conclusions

Altogether, our findings of genetic and non-genetic factors affecting the expression of COVID-19-related genes in bronchial epithelium provide essential insights for understanding inter-individual variation of COVID-19 and developing therapeutic targets for COVID-19.

## Supplementary Information

**Additional file 1.** Supplementary Methods.

**Additional file 2: Table S1.** Differential expression analysis of *ACE2* in relation to clinical variables (A) and genomic signatures (B) in SPIROMICS, SARP, and MAST. **Table S2.** Top 100 genes co-expressed with *ACE2* after adjustments in SPIROMICS (A), SARP (B), and MAST (C). The genes in the IL-17 signature are highlighted in yellow. **Table S3.** Canonical pathway gene sets based on differentially downregulated genes between SARS-CoV-2 infection and other viral illness using the Ingenuity Pathway Analysis canonical pathway function. **Table S4.** Association between canonical pathway gene sets from Table S3 and comorbidities in SPIROMICS (A), SARP (B), and MAST (C). Leading edge genes are enriched in association with the given comorbidity. **Table S5.** Canonical pathway gene sets based on genes enriched in association with each comorbidity using the Ingenuity Pathway Analysis canonical pathway function. A – cardiovascular condition in SPIROMICS, B – hypertension in SPIROMICS, C – obesity in SPIROMICS, D - hypertension in SARP, E – obesity in SARP. **Table S6.** COVID-19-related genes from Blanco-Melo et al. 2020, Gassen et al. 2020, Gordon et al. 2020, Hoffmann et al. 2020, Wang et al. 2020, and COVID-19 Cell Atlas. **Table S7.** Summary statistics of eQTL mapping in bronchial epithelium in SPIROMICS, including eQTL effect sizes, and lookup analysis from GTEx and eQTLGen Consortium. **Table S8.** Lookup of COVID-19-related genes with *cis*-eQTLs in bronchial epithelium from GTEx v8. Effect size measured as allelic fold change (log2) is given for every gene with FDR < 0.05 in GTEx v8 and its lead eQTL, or set to NA otherwise. **Table S9.** Pathway analysis of 492 eGenes from SPIROMICS not tested in GTEx Lung. **Table S10.** pheWAS of eQTLs for COVID-19-related genes in bronchial epithelium with Phenoscanner v2. **Table S11.** pheWAS of eQTLs for COVID-19-related genes in bronchial epithelium in (A) non-Hispanic White individuals (*N* = 1980) and (B) Hispanic and non-Hispanic, non-White individuals (*N* = 696) in SPIROMICS for 20 phenotypes. **Table S12.** Results of the colocalization analysis of the eQTLs in bronchial epithelium and COVID-19-relevant phenotypes.

**Additional file 3: Figure S1.** Associations between *ACE2* gene expression and COPD, asthma, steroid use, and race. **Figure S2.** Associations between *ACE2* gene expression and obesity**. Figure S3.** Associations between *ACE2* gene expression and hypertension, and use of antihypertensives**. Figure S4.** Associations between age and *ACE2* gene expression, and age and differential *ACE2* exon usage. **Figure S5.** Associations between age and smoking status, hypertension, sex, and BMI in SPIROMICS. **Figure S6.** COVID-19 and other viral illness related gene set enrichment analyses in association with comorbidities in SPIROMICS, SARP, and MAST. **Figure S7.** Regulatory genetic effects of *ACE2* and *TMPRSS2*, and the effect of smoking on *TMPRSS2*. **Figure S8.** Associations between COVID-19-related genes and comorbidities. **Figure S9.** Replication of *cis*-eQTLs in GTEx. **Figure S10.** Regulatory genetic effects of the candidate genes in the chr3 cluster associated with COVID-19. **Figure S11.** PheWAS associations for the 44 out of 108 lead *cis*-eQTLs associated with COVID-19-related genes with Phenoscanner v2.

**Additional file 4: Supplementary Note.** NHLBI Trans-Omics for Precision Medicine (TOPMed) Consortium Banner Authorship List.

## Data Availability

The RNA-seq data for SPIROMICS and SARP are deposited to dbGaP at accessions phs001119.v1.p1 and phs001446, respectively. While awaiting data release via dbGaP, investigators may contact the corresponding authors or the SPIROMICS and SARP studies at https://www.spiromics.org/spiromics/ and http://www.severeasthma.org/home.html to discuss gaining access to the data via the ancillary study mechanism for these studies. MAST RNA-seq data are available at Gene Expression Omnibus (GEO) (accession number GSE67472 [80]). TOPMed WGS freeze 9 data for the SPIROMICS cohort will be available at dbGaP under accession number phs001927. Full eQTL summary statistics for the 496 COVID-19-related genes generated during the current study can be downloaded from the GitHub repository at https://github.com/LappalainenLab/spiromics-covid19-eqtl/tree/master/eqtl/summary_stats [81]. eQTL mapping analyses code has been deposited to the GitHub repository at https://github.com/LappalainenLab/spiromics-covid19-eqtl [82].

## Abbreviations

SARS-CoV-2: Severe acute respiratory syndrome coronavirus 2
COVID-19: Coronavirus disease 2019
SPIROMICS: SubPopulations and InteRmediate Outcome Measures In COPD Study
COPD: Chronic obstructive pulmonary disease
SARP: Severe Asthma Research Program
MAST: Mechanisms of ASThma Study
eQTL: Expression quantitative trait locus
pheWAS: Phenome-wide association study
RNA-seq: RNA-sequencing
WGS: Whole genome sequencing
TOPMed: Trans-Omics for Precision Medicine
GTEx: Genotype-Tissue Expression
FEV1: Forced expiratory volume in 1 s
ERS/ATS: European Respiratory Society/American Thoracic Society
QC: Quality control
dACE2: Truncated *ACE2* transcript
FDR: False discovery rate
MAF: Minor allele frequency
TSS: Transcription start site
eGene: Gene with statistically significant eQTL
aFC: Allelic fold change
BMI: Body mass index
GWAS: Genome-wide association study
LD: Linkage disequilibrium
PP4: Posterior support for colocalization in coloc, defined as posterior probability for observing an association with both traits driven by a shared causal variant (hypothesis four)
FC: Fold change
ARB: Angiotensin receptor blockers
GSEA: Gene set enrichment analysis
IPA: Ingenuity Pathway Analysis
EFO: Experimental factor ontology
ISG: Interferon stimulated genes
