# Supplementary material for: Genetic and non-genetic factors affecting the expression of COVID-19-relevant genes in the large airway epithelium
Source: Genome Med. 2021 Apr 21;13:66. doi: 10.1186/s13073-021-00866-2 (PMC8059115; doi:10.1186/s13073-021-00866-2)
Supplement: Supplementary file 1 — Additional file 1. Supplementary Methods. [file 13073_2021_866_MOESM1_ESM.pdf]

Supplementary Methods for

Genetic and non-genetic factors affecting the expression of COVID-19 relevant genes in the large airway epithelium

Silva Kasela, Victor E Ortega, Molly Martorella, Suresh Garudadri, Jenna Nguyen, Elizabeth Ampleford, Anu Pasanen, Srilaxmi Nerella, Kristina L Buschur, Igor Z Barjaktarevic, R Graham Barr, Eugene R Bleecker, Russell P Bowler, Alejandro P Comellas, Christopher B Cooper, David J Couper, Gerard J Criner, Jeffrey L Curtis, MeiLan K Han, Nadia N Hansel, Eric A Hoffman[16], Robert J Kaner, Jerry A Krishnan, Fernando J Martinez, Merry-Lynn N McDonald, Deborah A Meyers, Robert Paine 3rd, Stephen P Peters, Mario Castro, Loren C Denlinger, Serpil C Erzurum, John V Fahy, Elliot Israel, Nizar N Jarjour, Bruce D Levy, Xingnan Li, Wendy C Moore, Sally E Wenzel, Joe Zein, NHLBI SubPopulations and InteRmediate Outcome Measures In COPD Study (SPIROMICS), NHLBI Trans-Omics for Precision Medicine (TOPMed) Consortium, Charles Langelier, Prescott G Woodruff, Tuuli Lappalainen[†], Stephanie A Christenson[†]

†These authors contributed equally to this work.

Differential expression analysis of *ACE2* in relation to host/environmental factors

Visualization and analyses of single gene and gene signature analyses were done using RLE normalized and COMBAT batch corrected gene expression from the DESeq2 and SVA packages in R. Linear regression models were fitted to evaluate associations between *ACE2* expression (based on normalized count) and clinical variables. In SPIROMICS unadjusted models were evaluated along with models adjusted for potential confounders including smoking status, age, sex, body mass index (BMI), and race, as appropriate. For analyses of hypertension, adjusted analyses included anti-hypertensives as a covariate, in addition to sex, age, smoking status and race. In SARP adjusted analyses included covariates for asthma, steroid use (inhaled steroids alone or inhaled plus oral steroids), age, sex, race, and BMI, as appropriate. Analyses of hypertension were done only in participants with asthma (due to availability of data), but adjustments for sex, age, BMI, and race were still done. In MAST analyses were adjusted for age, sex, asthma disease status, race, and BMI.

Differential exon usage

Following alignment, we indexed and sliced the SPIROMICS BAM files to include 51.6 kb of the *ACE2* genomic region (chrX:15,556,393-15,608,016 in the hg38 genome build) using samtools [1]. GTF files were manually curated to include the three exons that contribute to differential isoform expression of *ACE2* [2]. Full length *ACE2* transcripts are generated from two independent first exons, Exons 1a and 1b, with Exon 1b shared between these transcripts. The truncated *ACE2* transcript (*dACE2*) that does not bind the SARS-CoV-2 virus but is associated with an interferon-stimulated gene response in experimental models originates from Exon 1c. Coordinates from the preprint manuscript by Onabajo et al. [2] were used to curate the GTF file: Exon 1a: chrX:15,601,956-15,602,158, Exon 1b: chrX:15,600,726-15,601,014, Exon 1c: chrX:15,580,281-15,580,420. The exons were counted using the ASpli package in R [3]. As per the ASpli and EdgeR package recommendations, raw exon counts were adjusted for overall gene counts to remove the signal from differential gene expression using the formula: (Exon Count in each sample * mean raw *ACE2* count)/raw *ACE2* gene count in that sample. To adjust for differences in sequencing depth between samples the transformed counts were then multiplied by the size factor variable generated by the DESeq2 package from the sequencing analysis. Linear models adjusting for batch were then used to analyze differences in exon usage in association with covariates of interest. The primary analysis was to evaluate whether *ACE2* exon 1c differential usage was associated with increases in our interferon-stimulated gene signature. In secondary analyses we determined whether clinical covariates were associated with differential exon 1c

usage. Interpretation of differential exon usage requires consideration of the necessary adjustment for variation in total transcript count. Thus, if overall ACE2 expression is decreased in association with an outcome, a differential increase in one exon adjusts the expression of that isoform away from the overall negative association, but does not necessarily mean that the isoform is not negatively associated with the outcome to a lesser extent.

Gene set enrichment analysis of expression changes induced by COVID-19

Differential expression and gene set enrichment analyses were done using the limma and FGSEA packages in R [4, 5]. Data underwent TMM normalization and the voom transformation followed by linear model fit with empirical Bayes moderation of the standard errors. We built COVID-19 relevant gene sets from publicly available differential gene expression data [6] from participants who underwent nasal/oropharyngeal swab sampling at the time of acute respiratory illness for COVID-19 diagnosis (94 participants with COVID-19, 41 with other viral illness, 103 with no virus identified, viruses identified by metagenomic sequencing analysis). Expression gene sets were built using the 100 genes most up- and downregulated in association with infection type. Biological pathway gene sets were built by inputting the genes differentially downregulated between SARS-CoV-2 infection and other viral illness ($P < 0.05$) into the Ingenuity Pathway Analysis canonical pathway function. The pathway assessments were limited to downregulated genes given the relationship between downregulated gene sets and comorbidities in the initial analyses of the 100 gene sets. Gene set enrichment analyses were then performed using FGSEA [5] and the CAMERA function [7] in limma against gene lists ranked by their log fold change differential expression in association with comorbid clinical risk factors. Barcode plots were made using CAMERA. Normalized enrichment scores for heatmaps were extracted from FGSEA (not available through CAMERA, but CAMERA and FGSEA statistical results were similar). As smoking so strongly influences gene expression, in SPIROMICS differential expression analyses input into GSEA algorithms to evaluate clinical factors such as obesity, hypertension, cardiovascular conditions, and sex were first done in former smokers only to limit the effect of smoking, adjusting for age, sex and BMI if appropriate. In sensitivity analyses, we repeated the analyses in all subjects, adjusting for smoking status as well and found similar results. As asthma and steroid use so strongly influence gene expression, in SARP differential expression analyses of these other clinical factors were limited to asthma participants on inhaled but not oral steroids. Secondary analyses included all asthma participants adjusting for steroid use, with similar findings. Findings were considered significant at $P < 0.05$ and false discovery rate (FDR) < 0.05 if multiple corrections were necessary. For **Additional File 3: Figure S8**, in which we evaluated

COVID-19-related genes identified by experimental data from the SARS-CoV-2 *ex vivo* infection of primary human bronchial epithelial cells [8] or thought to be proteins that interact with SARS-CoV-2 [9], we reported findings at the less stringent $P < 0.05$ as these analyses were hypothesis generating only.

COVID-19-related genes

We mined the growing body of COVID-19 related literature to identify host genes implicated in SARS-CoV-2 infection discovered using different analytical approaches. The following studies were used to compose a list of COVID-19 candidate genes: 1) Hoffmann et al. [10] that identified ACE2 as the receptor to be exploited by the SARS-CoV-2 for cellular entry, and proteases TMPRSS2 and cathepsin B/L both to be used by SARS-CoV-2 for S protein priming, whilst only TMPRSS2 is essential for viral entry and viral spread; 2) Gordon et al. [9] that identified 332 high-confidence SARS-CoV-2-human protein-protein interactions; 3) Blanco-Melo et al. [8] that explored the transcriptional response to SARS-CoV-2 *in vitro*, *ex vivo*, and *in vivo* models; 4) COVID-19 Cell Atlas (www.covid19cellatlas.org, accessed 20 May 2020) that highlights 17 genes including cathepsins and other viral receptors or receptor associated enzymes; 5) Gassen et al. [11] that showed the role of SARS-CoV-2 infection in restricting AMPK/mTORC1 activation and autophagy; 6) Wang et al. [12] that reported a mediating role of CD147 (also known as BSG) in SARS-CoV-2 viral invasion.

To narrow the list of differentially expressed genes following SARS-CoV-2 infection from Blanco-Melo et al. [8], we focused on the results from the *ex vivo* infection of primary human bronchial epithelial cells. To include in our candidate list, we chose genes that 1) have adjusted P-value < 0.05 in the differential expression analysis from primary cells and either cell lines (Calu-3 or ACE2-expressing A549 cells, low-MOI infection; excluded genes with adjusted $P = 0$) or samples derived from COVID-19 patients, and 2) $\log_2$ fold change > 0.5 in absolute scale in primary cells and $\log_2$ fold change > 1 in absolute scale in the other experiment.

In total, we selected 514 candidate genes implicated in COVID-19 from six different sources. Of them, 496 genes were expressed in bronchial epithelium.

Expression quantitative trait mapping

Expression quantitative trait (eQTL) mapping was performed in 144 unrelated individuals from the SPIROMICS bronchoscopy sub-study with WGS genotype data from TOPMed and gene expression from bronchial epithelium profiled with RNA-seq following the analysis pipeline from

the Genotype-Tissue Expression (GTEx) Consortium [13]. Gene expression data was normalized as follows: 1) read counts were normalized between samples using TMM [14] with edgeR [15], 2) genes with TPM ≥ 0.1 and unnormalized read count ≥ 6 in at least 20% of samples were retained, 3) expression values were transformed using rank-based inverse normal transformation across samples.

Next, Probabilistic Estimation of Expression Residuals (PEER, [16]) was used to infer hidden determinants of variability in gene expression levels due to technical and biological sources. According to the optimization analysis for selection of PEERs by sample size to maximize *cis*-eGene discovery done in GTEx [13], 15 PEERs were chosen to be used as covariates in eQTL mapping together with 4 genotype PCs and sex imputed from genotype data.

To control population structure, principal component analysis (PCA) was conducted on post-variant QC genotype data from unrelated SPIROMICS individuals. More precisely, PCA was performed on a set of LD-independent autosomal biallelic single nucleotide polymorphisms from not long-range LD regions [17] with a call rate ≥ 99% and MAF ≥ 0.05 using smartpca from EIGENSTRAT (https://github.com/argriffing/eigensoft/tree/master/EIGENSTRAT), turning off outlier removal (option `-m 0`). LD pruning was performed using Plink 1.9 [18] based on pairwise genotypic correlation of 200 SNPs at a time, with a step of 100 SNPs, and using LD threshold of > 0.1 to remove one of a pair of SNPs (option `--indep-pairwise 200 100 0.1`). Top 4 PCs were chosen to be used to correct for population stratification. The top 4 PCs explained > 0.1% of the variance, and were associated with subpopulations inferred from 1000 Genomes Project using k-nearest neighbors clustering (F-test $P < 2\times10^{-10}$, adj $R^2 = 0.36 - 0.98$).

Cis-eQTL mapping was performed using tensorQTL [19] across 22,738 genes and 6,605,907 variants with MAF ≥ 0.05 and variant call rate ≥ 0.9. Window-size was set to 1 Mb from the transcription start site (TSS) of the gene according to the GENCODE version 33, 10,000 permutations were used to correct for multiple testing, and false discovery rate (FDR) < 0.05 was used to identify genes with statistically significant eQTLs (eGenes). We also used tensorQTL to map conditionally independent *cis*-eQTLs.

Lead *cis*-eQTL effect size was quantified as allelic fold change (aFC, [20]), ratio of expression of the haplotype carrying the alternative allele to expression of the haplotype carrying the reference allele of an eQTL. Gene expression data normalized with DESeq2 size factors [21] and $\log_2$-transformed were used as input together with the processed genotype VCF file. aFC was

calculated requiring at least 2 samples (`--min_samps 2`) and minimum 1 observation of each allele (`--min_alleles 1`), and adjusting for the same covariates as in *cis*-eQTL mapping. To calculate confidence intervals 100 bootstrap samples were used. aFC estimates that hit the absolute cap value ($\log_2(100)$) were set to missing.

Cell type interacting expression quantitative trait mapping

Firstly, we used xCell [22] to estimate 64 immune and stroma cell types from the gene expression signatures of bronchial epithelium. TPM expression matrix of 144 bronchial brush samples together with 30 samples from each tissue type from GTEx was uploaded to the UCSF xCell Webtool (https://xcell.ucsf.edu/). Then, the following linear regression model was used to map cell type interaction eQTLs (ieQTLs): p ~ g+i+g×i+C, where *p* is the phenotype vector (inverse normalized gene expression), *g* is the genotype vector, *i* is the cell type enrichment score from xCell (inverse normalized), *g*×*i* is the interaction term, and *C* is the covariates matrix as used in eQTL mapping. Cell types with a median xCell enrichment score > 0.05 were included in the analysis. There were 29 cell type signatures that met this criteria: B cells, basophils, CD4 TCM, CD4 TEM, CD8 naïve T cells, common lymphoid progenitor (CLP), common myeloid progenitor (CMP), class switched memory B cells, DCs, eosinophils, epithelial cells, HSCs, keratinocytes, M1 and M2 macrophages, monocytes, osteoblasts, plasma cells, preadipocytes, sebocytes, smooth muscle, TH2 cells, antigen-presenting, immature, classical and plasmacytoid dendritic cells, pro B cells, and microenvironment and immune scores. Mapping of ieQTLs was done using tensorQTL [19], and only variants within 1Mb of the TSS of each gene tested and with MAF > 0.1 in the samples belonging to the top and bottom halves of the distribution of cell type abundance were included. Regression coefficients and *P*-values were calculated for all terms in the model, and ieQTLs were identified by testing for the significance of the interaction term. Top nominal *P*-values for each gene were corrected for multiple testing at the gene level using eigenMT [23] as implemented in tensorQTL. Significance across genes was determined by adjusting eigenMT *P*-values using the Benjamini-Hochberg procedure with FDR of 0.05.

Replication of *cis*-eQTLs in GTEx

We performed replication of *cis*-eQTLs (gene-variant pairs) found from bronchial epithelium in the Genotype-Tissue Expression (GTEx) project v8 release [13]. Using *cis*-eQTL summary statistics across 49 tissues from GTEx, we calculated the proportion of true positives [24], π_1, to estimate the proportion of sharing of *cis*-eQTLs between bronchial epithelium and GTEx tissues. We

assessed the allelic direction of the *cis*-eQTLs from bronchial epithelium and GTEx tissues by calculating concordance rate, the proportion of gene-variant pairs with the same allelic direction. This comparison was restricted to *cis*-eQTLs with nominal *P*-value < $1x10^{-4}$ in the given GTEx tissue.

Next, we analyzed the replication and concordance measure as a function of sample size and median cell type enrichment scores for seven cell types [25]: adipocytes, epithelial cells, hepatocytes, keratinocytes, myocytes, neurons, and neutrophils. Tissues with median enrichment score > 0.1 were classified as being enriched for the given cell type. We used a two-sided Wilcoxon rank sum test to estimate the difference in replication and concordance estimates between tissues enriched or not enriched for the given cell type, and Spearman correlation coefficient to calculate correlation between sample size and the two concordance measures (π_1 and concordance rate).

cis-eQTLs not identified in previous large eQTL catalogs

To investigate the tissue-specificity of *cis*-eQTLs from bronchial epithelium, we performed gene-level lookup in GTEx v8 and eQTLGen Consortium [26]. We identified genes with significant regulatory effects in SPIROMICS (FDR < 0.05) that were tested in neither catalog.

Then, we used the functional profiling webtool g:GOSt (version e99_eg46_p14_f929183) from g:Profiler [27] to perform pathway analysis of the 492 significant eGenes in SPIROMICS not tested in GTEx v8 Lung. Method g:SCS was used for multiple testing correction corresponding to experiment-wide threshold of α = 0.05. Significant eGenes from SPIROMICS (n = 4,881) that have at least one annotation (option “Custom over annotated genes”) were used as a background in the enrichment test.

pheWAS of lead COVID-19 *cis*-eQTLs in SPIROMICS

We performed phenome-wide association studies (pheWAS) in 1,980 non-Hispanic White (NHW) and 696 individuals from other ethnic and racial groups from SPIROMICS for the 108 lead *cis*-eQTLs to evaluate for phenotypic associations with spirometric measures, cell count differentials, immunoglobulin concentrations, longitudinal exacerbation risk, self-reported asthma history, cardiovascular diseases, CT scan measures of emphysema (bilateral percentage lung density < -950HFU at total lung capacity), CT scan functional small airways disease (PRM-fSAD), and alpha1-antitrypsin concentrations (subgroup of 1,191 NHW and 396 from other racial/ethnic groups). PheWAS regression-based models were performed using PLINK 2/0 and included

principle components of ancestry, sex, BMI, age, and smoking pack-years. Models for CT scan measures also included site and height while alpha1-antitrypsin concentrations included c-reactive protein. CT scan measures, eosinophil counts, and IgE concentrations were log-transformed. Significance threshold was set for the number of eQTLs tested across phenotypes ($P < 4.63 \times 10^{-4}$).

Lookup of phenome-wide associations with PhenoScanner v2

PhenoScanner v2 [28, 29] was used to lookup phenotype associations for the *cis*-eQTL variants from large-scale genome-wide association studies (GWAS) with association P-value < 10^{-5}. We queried PhenoScanner database based on the rs IDs of the lead *cis*-eQTLs obtained from dbSNP version 151 (GRCh38p7, including also former rs ID to query). The phenoscanner R package (https://github.com/phenoscanner/phenoscanner) was used to perform the queries. Query results were filtered to keep one association for each of the variants per trait, preferring summary statistics from newer studies, studies with larger sample size, or based on UK Biobank data (GWAS round 1 results from the Neale Lab). Description of Experimental Factor Ontology (EFO) terms and classification to EFO broader categories were obtained from the GWAS Catalog or by manually searching EMBL-EBI EFO webpage (https://www.ebi.ac.uk/efo/).

The regulatory variants for *CEP250*, *FAR2*, and *TLE3* have phenotypic associations with both body height and pulmonary function test (PFT) measures from Phenoscanner. As GWAS analyses from the Neale Lab using UK Biobank data do not include height as a covariate in the model, we used the results of the lung function GWAS by Shrine et al. [30] to confirm if the suggestive signal for PFT trait has been observed before or rather seems to be an artefact of incomplete adjustment for height. Of note, Shrine et al. have discovered 279 lung functions signals in the meta-analysis of UK Biobank and the SpiroMeta Consortium. We looked up the nearest GWAS hits to the eQTL, and calculated LD between the variants in the African and European populations using LDpop [31] web tool.

Colocalization analysis

Multiple trait associations observed for a single variant do not necessarily translate into shared genetic causality. To assess evidence for shared causal variant of a *cis*-eQTL and a GWAS trait, we used the Bayesian statistical test for colocalization, coloc. We used the newer version of coloc [32] that allows conditioning and masking to overcome one single causal variant assumption (condmask branch of coloc from https://github.com/chr1swallace/coloc). We only tested

colocalization for loci where the eQTL had at least one phenotypic association based on the lookup analysis with Phenoscanner from the following EFO parent categories: hematological measurement, pulmonary function measurement, respiratory disease. From each of the smaller EFO categories, we chose one trait with the smallest *P*-value for which we were able to find summary statistics using GWAS Catalog REST API or among the Neale Lab GWAS round 2 results (http://www.nealelab.is/uk-biobank/). Coloc was run on a 500-kb region centered on the lead *cis*-eQTL (+/- 250 kb from the variant) with priors set to $p_1 = 10^{-4}$, $p_2 = 10^{-4}$, $p_3 = 5x10^{-6}$. We used the coloc.signals() function with `mode = iterative` and `method = mask` for GWAS traits with LD data from the 1000 Genomes Project to match the ancestry of the discovery population (*e.g.*, choosing CEU for LD if the discovery population is of European ancestry). We allowed for a maximum of three variants to mask, with an r^2 threshold of 0.01 to call two signals independent and *P*-value threshold of $1x10^{-5}$ to call the secondary signal significant. We used `method = single` for the eQTLs, because the corresponding eGenes did not have secondary independent signals. We prioritized eGenes with posterior probability for colocalization (PP4) > 0.5 as loci with evidence for colocalization.

References

1. Li H, Handsaker B, Wysoker A, Fennell T, Ruan J, Homer N, et al. The Sequence Alignment/Map format and SAMtools. Bioinformatics. 2009;25:2078–9.

2. Onabajo OO, Banday AR, Stanifer ML, Yan W, Obajemu A, Santer DM, et al. Interferons and viruses induce a novel truncated ACE2 isoform and not the full-length SARS-CoV-2 receptor. Nat Genet. 2020.

3. Mancini E, Rabinovich A, Iserte J, Yanovsky M, Chernomoretz A. ASpli: Analysis of alternative splicing using RNA-Seq. 2020.

4. Ritchie ME, Phipson B, Wu D, Hu Y, Law CW, Shi W, et al. limma powers differential expression analyses for RNA-sequencing and microarray studies. Nucleic Acids Res. 2015;43:e47.

5. Korotkevich G, Sukhov V, Sergushichev A. Fast gene set enrichment analysis. Preprint at bioRxiv https://doi.org/10.1101/060012. 2019.

6. Mick E, Kamm J, Pisco AO, Ratnasiri K, Babik JM, Calfee CS, et al. Upper airway gene expression differentiates COVID-19 from other acute respiratory illnesses and reveals suppression of innate immune responses by SARS-CoV-2. Preprint at medRxiv https://doi.org/10.1101/2020.05.18.20105171. 2020.

7. Wu D, Smyth GK. Camera: a competitive gene set test accounting for inter-gene correlation. Nucleic Acids Res. 2012;40:e133.

8. Blanco-Melo D, Nilsson-Payant BE, Liu W-C, Uhl S, Hoagland D, Møller R, et al. Imbalanced Host Response to SARS-CoV-2 Drives Development of COVID-19. Cell. 2020;181:1036-1045.e9.

9. Gordon DE, Jang GM, Bouhaddou M, Xu J, Obernier K, White KM, et al. A SARS-CoV-2 protein interaction map reveals targets for drug repurposing. Nature. 2020;583:459–68.

10. Hoffmann M, Kleine-Weber H, Schroeder S, Krüger N, Herrler T, Erichsen S, et al. SARS-CoV-2 Cell Entry Depends on ACE2 and TMPRSS2 and Is Blocked by a Clinically Proven Protease Inhibitor. Cell. 2020;181:271-280.e8.

11. Gassen NC, Papies J, Bajaj T, Dethloff F, Emanuel J, Weckmann K, et al. Analysis of SARS-CoV-2-controlled autophagy reveals spermidine, MK-2206, and niclosamide as putative antiviral therapeutics. Preprint at bioRxiv https://doi.org/10.1101/2020.04.15.997254. 2020.

12. Wang K, Chen W, Zhou Y-S, Lian J-Q, Zhang Z, Du P, et al. SARS-CoV-2 invades host cells via a novel route: CD147-spike protein. Preprint at bioRxiv https://doi.org/10.1101/2020.03.14.988345. 2020.

13. The GTEx Consortium. The GTEx Consortium atlas of genetic regulatory effects across human tissues. Science. 2020;369:1318–30.

14. Robinson MD, Oshlack A. A scaling normalization method for differential expression analysis of RNA-seq data. Genome Biol. 2010;11:R25.

15. Robinson MD, McCarthy DJ, Smyth GK. edgeR: a Bioconductor package for differential expression analysis of digital gene expression data. Bioinformatics. 2010;26:139–40.

16. Stegle O, Parts L, Durbin R, Winn J. A Bayesian framework to account for complex non-genetic factors in gene expression levels greatly increases power in eQTL studies. PLoS Comput Biol. 2010;6:e1000770.

17. Price AL, Weale ME, Patterson N, Myers SR, Need AC, Shianna KV, et al. Long-range LD can confound genome scans in admixed populations. Am J Hum Genet. 2008;83:132–5.

18. Purcell S, Neale B, Todd-Brown K, Thomas L, Ferreira MAR, Bender D, et al. PLINK: a tool set for whole-genome association and population-based linkage analyses. Am J Hum Genet.

2007;81:559–75.

19. Taylor-Weiner A, Aguet F, Haradhvala NJ, Gosai S, Anand S, Kim J, et al. Scaling computational genomics to millions of individuals with GPUs. Genome Biol. 2019;20:228.

20. Mohammadi P, Castel SE, Brown AA, Lappalainen T. Quantifying the regulatory effect size of cis-acting genetic variation using allelic fold change. Genome Res. 2017;27:1872–84.

21. Love MI, Huber W, Anders S. Moderated estimation of fold change and dispersion for RNA-seq data with DESeq2. Genome Biol. 2014;15:550.

22. Aran D, Hu Z, Butte AJ. xCell: digitally portraying the tissue cellular heterogeneity landscape. Genome Biol. 2017;18:220.

23. Davis JR, Fresard L, Knowles DA, Pala M, Bustamante CD, Battle A, et al. An Efficient Multiple-Testing Adjustment for eQTL Studies that Accounts for Linkage Disequilibrium between Variants. Am J Hum Genet. 2016;98:216–24.

24. Storey JD, Tibshirani R. Statistical significance for genomewide studies. Proc Natl Acad Sci U S A. 2003;100:9440–5.

25. Kim-Hellmuth S, Aguet F, Oliva M, Muñoz-Aguirre M, Kasela S, Wucher V, et al. Cell type–specific genetic regulation of gene expression across human tissues. Science. 2020;369. doi:10.1126/science.aaz8528.

26. Võsa U, Claringbould A, Westra H-J, Bonder MJ, Deelen P, Zeng B, et al. Unraveling the polygenic architecture of complex traits using blood eQTL metaanalysis. Preprint at bioRxiv https://doi.org/10.1101/447367. 2018.

27. Raudvere U, Kolberg L, Kuzmin I, Arak T, Adler P, Peterson H, et al. g:Profiler: a web server for functional enrichment analysis and conversions of gene lists (2019 update). Nucleic Acids Res. 2019;47:W191–8.

28. Staley JR, Blackshaw J, Kamat MA, Ellis S, Surendran P, Sun BB, et al. PhenoScanner: a database of human genotype-phenotype associations. Bioinformatics. 2016;32:3207–9.

29. Kamat MA, Blackshaw JA, Young R, Surendran P, Burgess S, Danesh J, et al. PhenoScanner V2: an expanded tool for searching human genotype-phenotype associations. Bioinformatics. 2019;35:4851–3.

30. Shrine N, Guyatt AL, Erzurumluoglu AM, Jackson VE, Hobbs BD, Melbourne CA, et al. New genetic signals for lung function highlight pathways and chronic obstructive pulmonary disease associations across multiple ancestries. Nat Genet. 2019;51:481–93.

31. Alexander TA, Machiela MJ. LDpop: an interactive online tool to calculate and visualize geographic LD patterns. BMC Bioinformatics. 2020;21:14.

32. Wallace C. Eliciting priors and relaxing the single causal variant assumption in colocalisation analyses. PLoS Genet. 2020;16:e1008720.
