# Supplementary material for: Genetic and non-genetic factors affecting the expression of COVID-19-relevant genes in the large airway epithelium
Source: Genome Med. 2021 Apr 21;13:66. doi: 10.1186/s13073-021-00866-2 (PMC8059115; doi:10.1186/s13073-021-00866-2)
Supplement: Supplementary file 3 — Additional file 3: Figure S1. Associations between ACE2 gene expression and COPD, asthma, steroid use, and race. Figure S2. Associations between ACE2 gene expression and obesity. Figure S3. Associations between ACE2 gene expression and hypertension, and use of antihypertensives. Figure S4. Associations between age and ACE2 gene expression, and age and differential ACE2 exon usage. Figure S5. Associations between age and smoking status, hypertension, sex, and BMI in SPIROMICS. Figure S6. COVID-19 and other viral illness related gene set enrichment analyses in association with comorbidities in SPIROMICS, SARP, and MAST. Figure S7. Regulatory genetic effects of ACE2 and TMPRSS2, and the effect of smoking on TMPRSS2. Figure S8. Associations between COVID-19-related genes and comorbidities. Figure S9. Replication of cis-eQTLs in GTEx. Figure S10. Regulatory genetic effects of the candidate genes in the chr3 cluster associated with COVID-19. Figure S11. PheWAS associations for the 44 out of 108 lead cis-eQTLs associated with COVID-19-related genes with Phenoscanner v2. [file 13073_2021_866_MOESM3_ESM.pdf]

Supplementary Figures for

Genetic and non-genetic factors affecting the expression of COVID-19 relevant genes in the large airway epithelium

Silva Kasela, Victor E Ortega, Molly Martorella, Suresh Garudadri, Jenna Nguyen, Elizabeth Ampleford, Anu Pasanen, Srilaxmi Nerella, Kristina L Buschur, Igor Z Barjaktarevic, R Graham Barr, Eugene R Bleecker, Russell P Bowler, Alejandro P Comellas, Christopher B Cooper, David J Couper, Gerard J Criner, Jeffrey L Curtis, MeiLan K Han, Nadia N Hansel, Eric A Hoffman[16], Robert J Kaner, Jerry A Krishnan, Fernando J Martinez, Merry-Lynn N McDonald, Deborah A Meyers, Robert Paine 3rd, Stephen P Peters, Mario Castro, Loren C Denlinger, Serpil C Erzurum, John V Fahy, Elliot Israel, Nizar N Jarjour, Bruce D Levy, Xingnan Li, Wendy C Moore, Sally E Wenzel, Joe Zein, NHLBI SubPopulations and InteRmediate Outcome Measures In COPD Study (SPIROMICS), NHLBI Trans-Omics for Precision Medicine (TOPMed) Consortium, Charles Langelier, Prescott G Woodruff, Tuuli Lappalainen[†], Stephanie A Christenson[†]

†These authors contributed equally to this work.

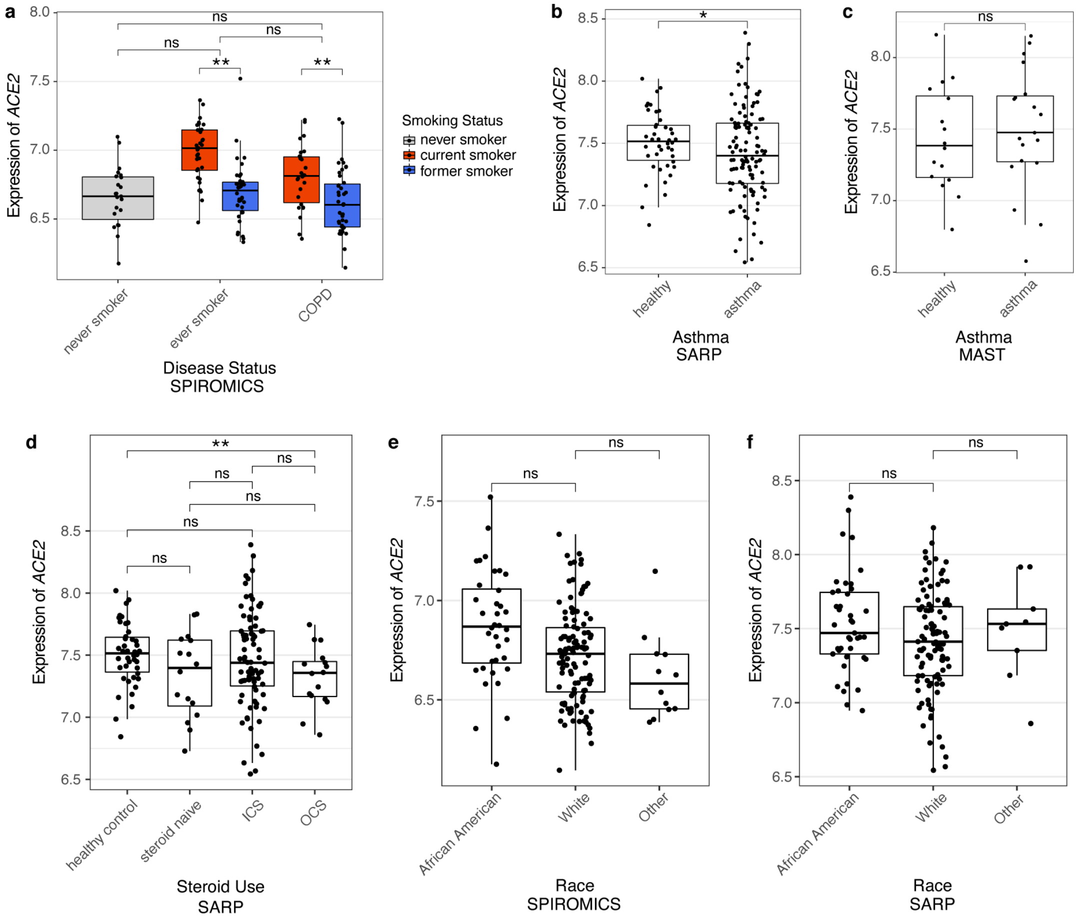

Figure S1. Associations between *ACE2* gene expression and COPD, asthma, steroid use, and race. a, Box plots showing *ACE2* log$_2$ gene expression in association with COPD in SPIROMICS. While current smoking significantly affected *ACE2* log$_2$ gene expression, there was no association with COPD after adjusting for smoking status ($P > 0.05$ for never smokers compared to current/former smokers with COPD and $P > 0.05$ for current/former smokers without COPD vs current/former smokers with COPD). **b-c**, Box plots showing *ACE2* log$_2$ gene expression in association with asthma in SARP and MAST. In SARP, *ACE2* levels were slightly lower in asthma compared to healthy controls. There was no significant association in MAST. **d**, If stratified by any steroid use in *SARP*, *ACE2* levels were significantly lower in asthmatics on oral but not inhaled steroids compared to healthy controls. ICS - inhaled steroids, OCS - oral steroids. **e-f**, Box plots showing *ACE2* log$_2$ gene expression in association with race in SPIROMICS and SARP. While African American race was associated with increased *ACE2* expression in both SPIROMICS (P = 0.002 before adjustments) and SARP (P = 0.042 before adjustment), this association was non-significant after adjusting for covariates. *P*-values: ****<0.0001, ***<0.001, **<0.01, *<0.05, ns=not significant in linear models adjusted for covariates. The boxes denote the interquartile range, the center line denotes the median, and whiskers denote the interquartile range×1.5.

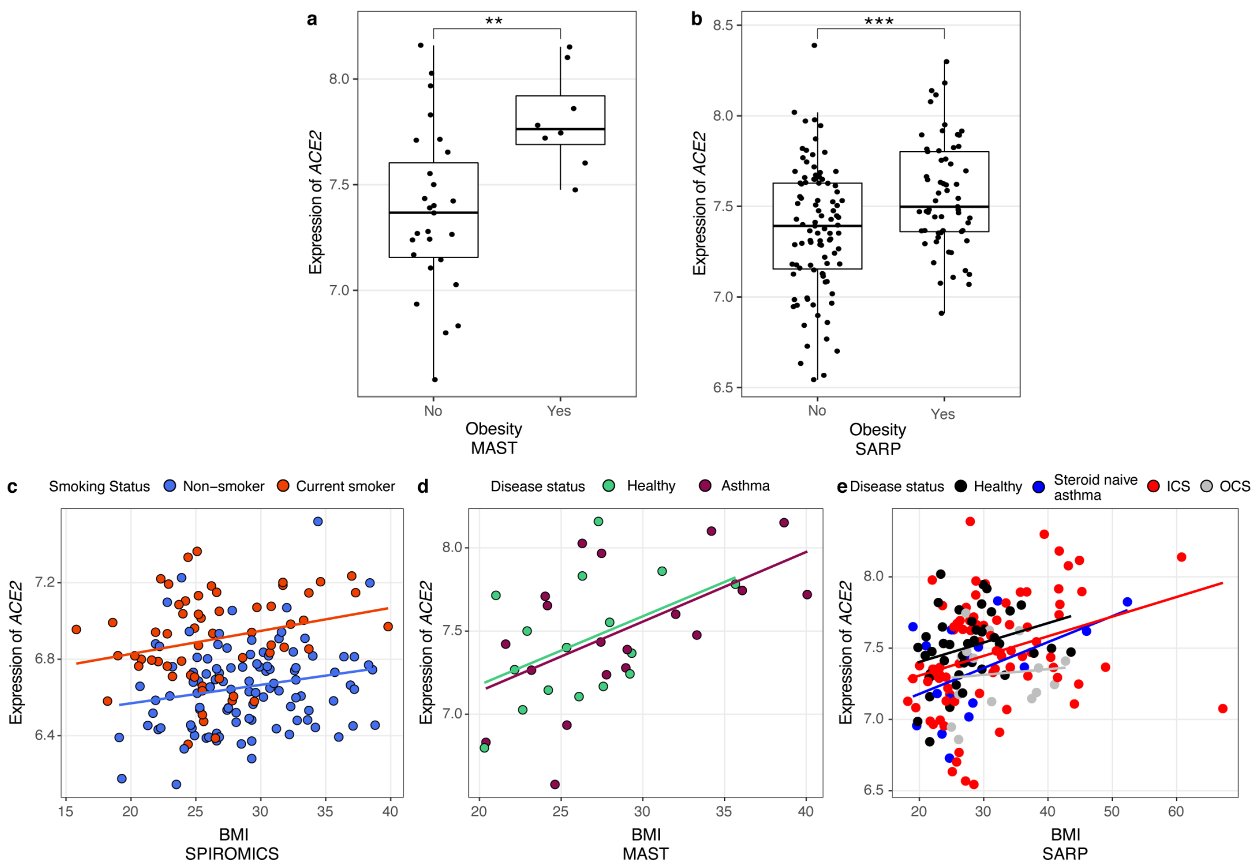

Figure S2. Associations between *ACE2* gene expression and obesity. a-b, Box plots showing *ACE2* $\log_2$ gene expression increased with obesity across MAST and SARP. *P*-values: ****<0.0001, ***<0.001, **<0.01, *<0.05, ns=not significant in linear models adjusted for covariates. The boxes denote the interquartile range, the center line denotes the median, and whiskers denote the interquartile range×1.5. **c-e**, Scatterplots showing *ACE2* $\log_2$ gene expression also increased with increasing body mass index as a continuous variable. Plots show that this association was robust smoking status in SPIROMICS (**c**), asthma status in MAST (**d**) and SARP (**e**). ICS - inhaled steroids, OCS - oral steroids.

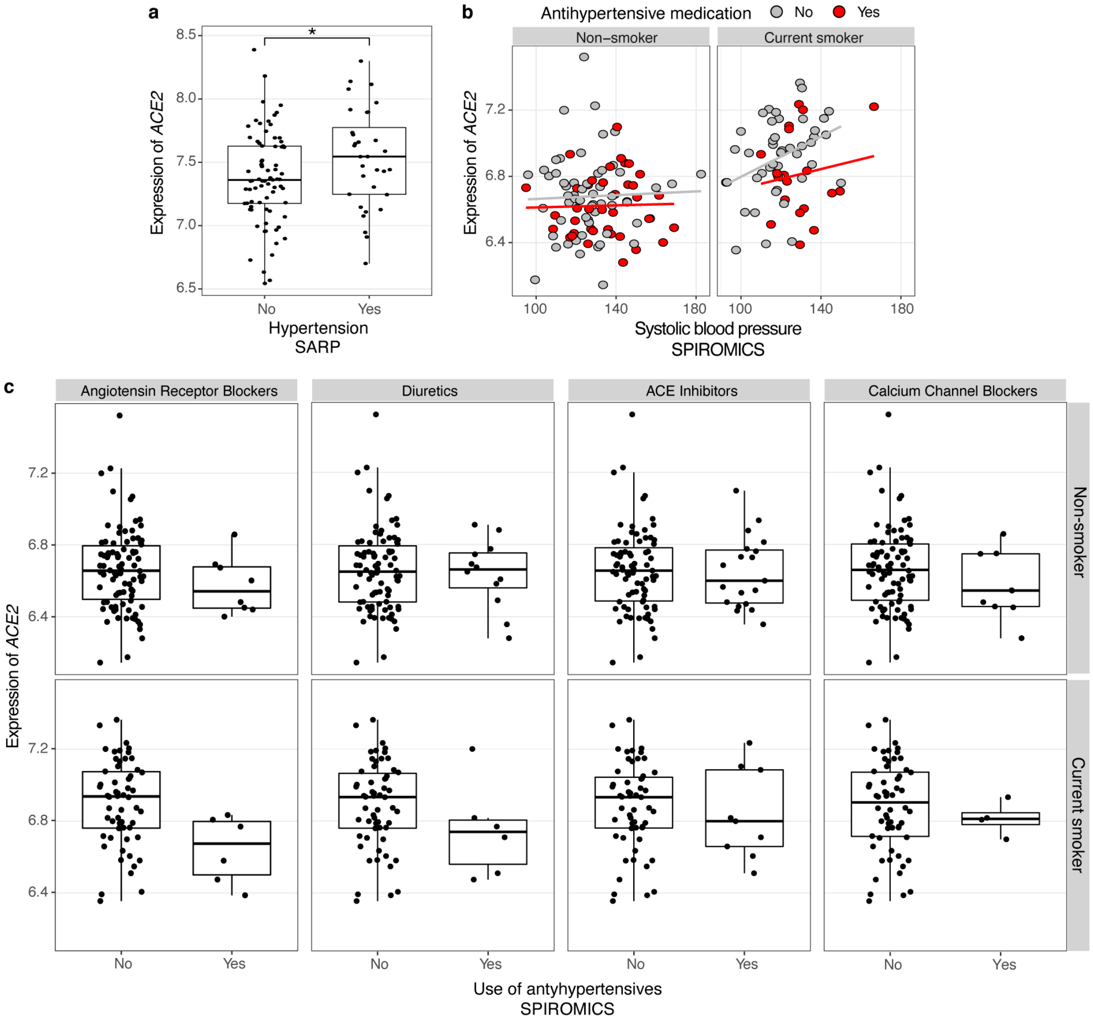

Figure S3. Associations between *ACE2* gene expression and hypertension, and use of antihypertensives. a, Box plots showing *ACE2* $\log_2$ gene expression increased with hypertension in SARP, data not collected in MAST. *P*-values: ****<0.0001, ***<0.001, **<0.01, *<0.05, ns=not significant in linear models adjusted for covariates. **b**, Scatterplots showing *ACE2* $\log_2$ gene expression also increased when considering systolic blood pressure as a continuous variable among current smokers. **c**, Box plots showing *ACE2* $\log_2$ gene expression in association with the use of antihypertensives by antihypertensive class (Angiotensin Receptor Blockers, Diuretics, ACE Inhibitors, Calcium Channel Blockers, respectively). Use of Angiotensin Receptor Blockers (ARBs) and Diuretics were associated with lower *ACE2* levels in unadjusted model (P = 0.021) and adjusted model in current smokers only (P = 0.023). In **a** and **c**, the boxes denote the interquartile range, the center line denotes the median, and whiskers denote the interquartile range×1.5.

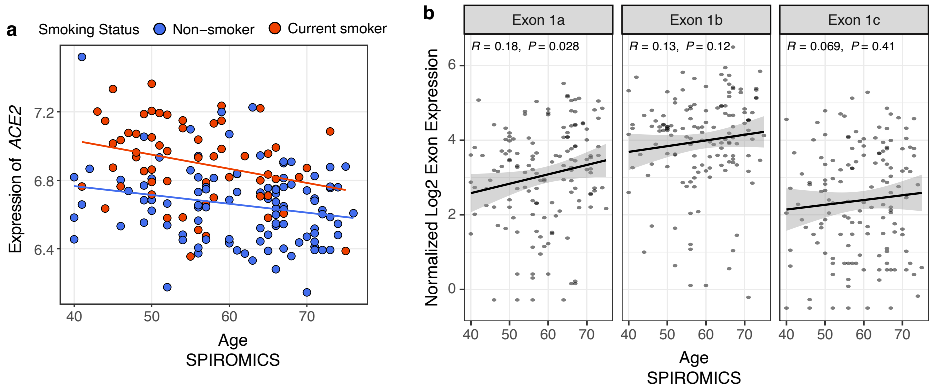

Figure S4. Associations between age and *ACE2* gene expression, and age and differential *ACE2* exon usage. a, In SPIROMICS, *ACE2* gene expression was modestly decreased in association with advancing age, but this finding was not replicated in either MAST or SARP (**Additional file 2: Table S1A**). **b**, Scatterplots showing increased Exon 1a usage with advancing age that was not observed for Exon 1b and 1c.

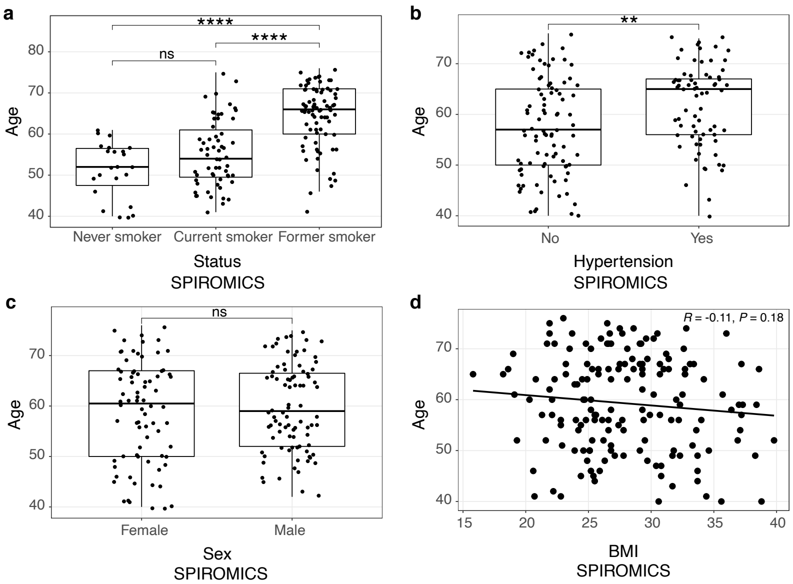

Figure S5. Associations between age and smoking status, hypertension, sex, and BMI in SPIROMICS. a-c, Boxplots showing that older age was associated with former smoking (**a**) and hypertension (**b**) but not sex (**c**). *P*-values: ****<0.0001, ***<0.001, **<0.01, *<0.05, ns=not significant in linear models adjusted for covariates. The boxes denote the interquartile range, the center line denotes the median, and whiskers denote the interquartile range×1.5. **d**, Scatterplot showing age and BMI were not significantly correlated.

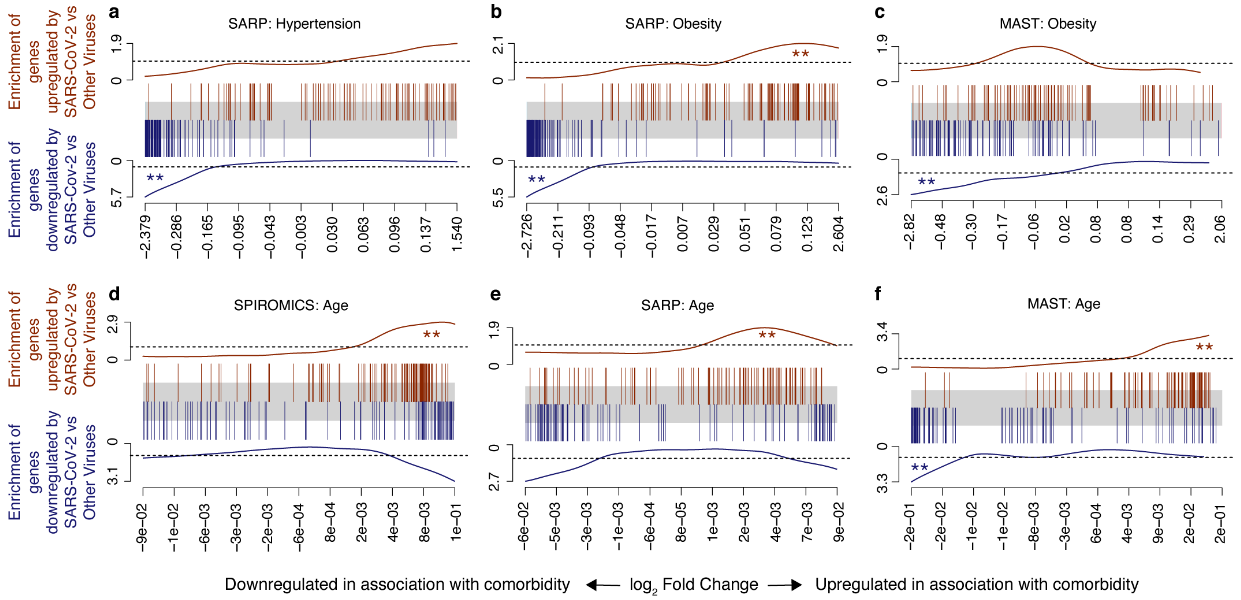

Figure S6. COVID-19 and other viral illness related gene set enrichment analyses in association with comorbidities in SPIROMICS, SARP, and MAST. a-f, Barcode plots in which the vertical lines represent the 100 genes most upregulated (red) or downregulated (blue) in nasal/oropharyngeal swab samples obtained from patients with COVID-19 as compared other viruses at the time of diagnosis of an acute upper respiratory infection (Mick et al. [1]). These gene sets are plotted against log fold gene expression changes arranged from most downregulated to most upregulated with a given comorbidity (horizontal grey bar). Lines above (red) and below (blue) the bar represent the running sum statistic with a significant finding indicated when the line crosses the dashed line at either end of the plot. Comorbidities included in the analyses are hypertension in SARP (**a**), obesity in SARP and MAST (**b-c**), age in SPIROMICS, SARP, and MAST (**d-f**).

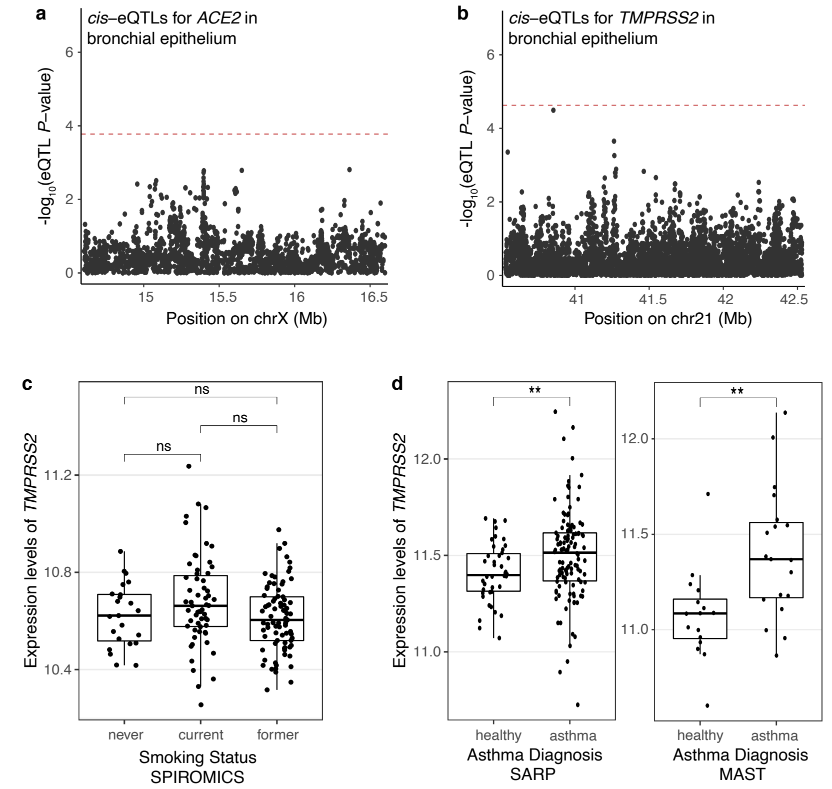

Figure S7. Regulatory genetic effects of *ACE2* and *TMPRSS2*, and the effect of smoking on *TMPRSS2*. a-b, Regional association plot illustrating no statistically significant regulatory effects on the gene expression levels of *ACE2* (**a**) and *TMPRSS2* (**b**). Each data point represents a genetic variant in the +/- 1Mb *cis*-window of the transcription start site of the given gene. Red dashed line denotes the gene-level significance threshold for the given gene. **c**, Box plot showing *TMPRSS2* $\log_2$ gene expression in association with smoking in SPIROMICS. Smoking had a more modest effect on the expression of *TMRPSS2* and was only significantly increased in current smokers when compared to never and current smokers overall ($P = 0.026$) but not when stratified by former and never smokers, as shown in the box plot. ns=not significant in linear models adjusted for covariates. **d**, Box plot showing *TMPRSS2* $\log_2$ gene expression in association with asthma in SARP (left) and MAST (right). Expression levels of *TMPRSS2* were higher in asthmatic than healthy controls ($P = 0.038$ in SARP, $P = 0.0028$ in MAST). In **c** and **d,** the boxes denote the interquartile range, the center line denotes the median, and whiskers denote the interquartile range×1.5.

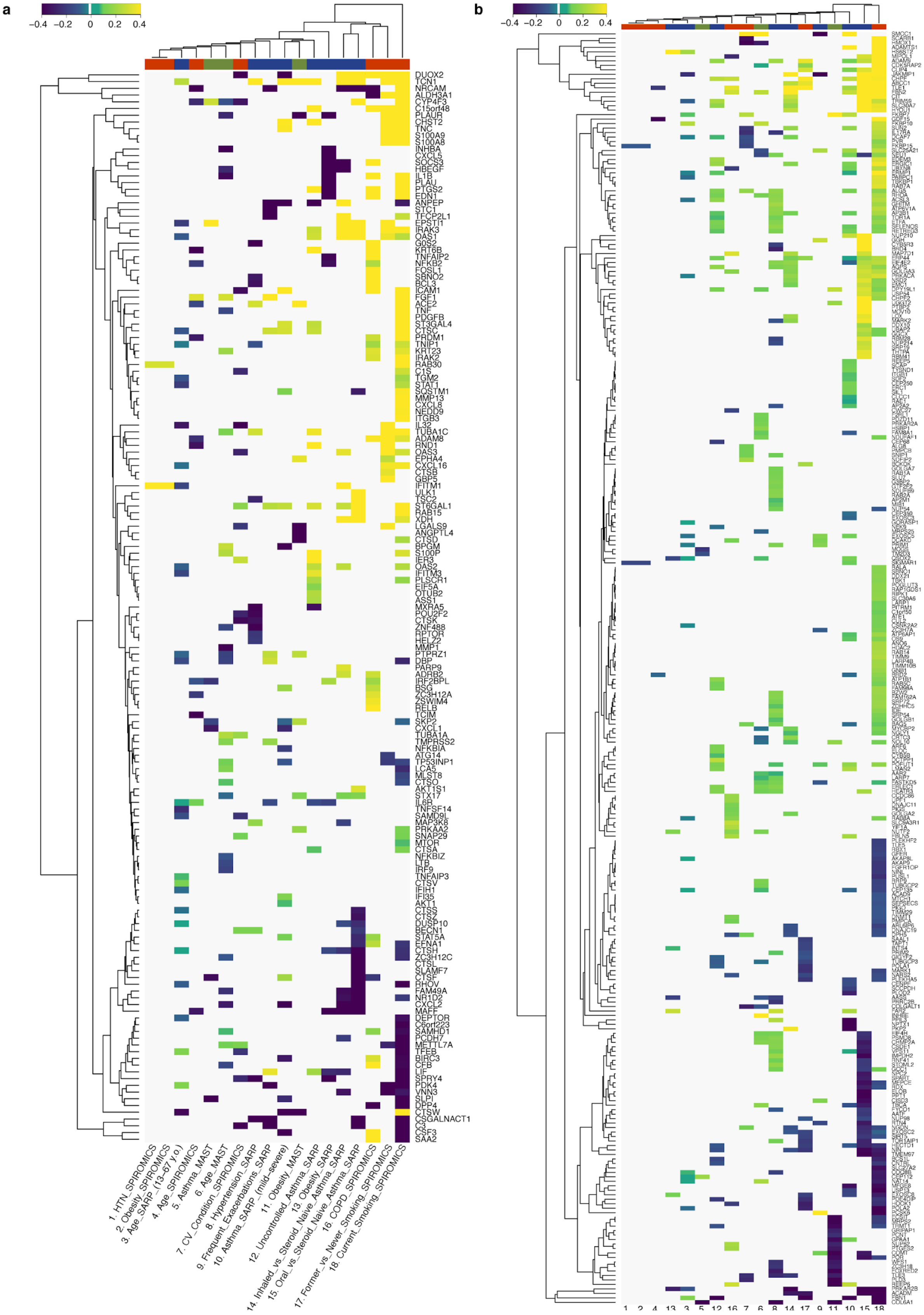
a
−0.4 −0.2 0 0.2 0.4
DUOX2
TCN1
NRCAM
ALDH3A1
CYP4F3
C15orf48
PLAUR
CHST2
TNC
S100A9
S100A8
INHBA
CXCL5
SOCS3
HBEGF
IL1B
PLAU
PTGS2
EDN1
ANPEP
STC1
TFCP2L1
EPSTI1
IRAK3
OAS1
G0S2
KRT6B
TNFAIP2
NFKB2
FOSL1
SBNO2
BCL3
ICAM1
FGF1
ACE2
TNF
PDGFB
ST3GAL4
CTSC
PRDM1
TNIP1
KRT23
IRAK2
RAB30
C1S
TGM2
STAT1
SQSTM1
MMP13
CXCL8
NEDD9
ITGB3
IL32
TUBA1C
ADAM8
RND1
OAS3
EPHA4
CXCL16
CTSB
GBP5
IFITM1
ULK1
TSC2
ST6GAL1
RAB15
XDH
LGALS9
ANGPTL4
CTSD
BPGM
S100P
IER3
OAS2
IFITM3
PLSCR1
EIF5A
OTUB2
ASS1
MXRA5
POU2F2
CTSK
ZNF488
RPTOR
HELZ2
MMP1
PTPRZ1
DBP
PARP9
ADRB2
IRF2BPL
BSG
ZC3H12A
ZSWIM4
RELB
TCIM
SKP2
CXCL1
TUBA1A
TMPRSS2
NFKBIA
ATG14
TP53INP1
LCA5
MLST8
CTSO
AKT1S1
STX17
IL6R
TNFSF14
SAMD9L
MAP3K8
PRKAA2
SNAP29
MTOR
CTSA
NFKBIZ
LTB
IRF9
TNFAIP3
CTSV
IFIH1
IFI35
AKT1
CTSS
CTSZ
DUSP10
BECN1
STAT5A
EFNA1
CTSH
ZC3H12C
CTSL
SLAMF7
CTSF
RHOV
FAM49A
NR1D2
CXCL2
MAFF
DEPTOR
C6orf223
SAMHD1
PCDH7
METTL7A
TFEB
BIRC3
CFB
LIF
SPRY4
PDK4
VNN3
SLPI
DPP4
CTSW
CSGALNACT1
C3
CSF3
SAA2
1. HTN_SPIROMICS
2. Obesity_SPIROMICS
3. Age_SARP_(13–67 y.o.)
4. Age_SPIROMICS
5. Asthma_MAST
6. Age_MAST
7. CV_Condition_SPIROMICS
8. Hypertension_SARP
9. Frequent_Exacerbations_SARP
10. Asthma_SARP_(mild–severe)
11. Obesity_MAST
12. Uncontrolled_Asthma_SARP
13. Obesity_SARP
14. Inhaled_vs_Steroid_Naive_Asthma_SARP
15. Oral_vs_Steroid_Naive_Asthma_SARP
16. COPD_SPIROMICS
17. Former_vs_Never_Smoking_SPIROMICS
18. Current_Smoking_SPIROMICS
b
−0.4 −0.2 0 0.2 0.4
1 2 4 13 3 5 12 16 7 6 8 14 17 9 11 10 15 18

Figure S8. Associations between COVID-19-related genes and comorbidities. **a,** COVID-19-related genes identified in experimental models with primary human bronchial epithelial cells from Blanco-Melo et al. [4] are shown in the heatmap (rows) plotted against comorbidities (columns) with genes differentially down- and upregulated in association with that comorbidity at P-value < 0.05 indicated in blue and yellow, respectively. **b,** Similar heatmap showing genes identified as potentially interacting with the SARS-CoV-2 virus by protein-protein interaction analyses (rows) from Gordon et al. [5]. All genes not associated at P-value < 0.05 were shrunk to zero (white). Euclidean distance with average linkage was used for clustering.

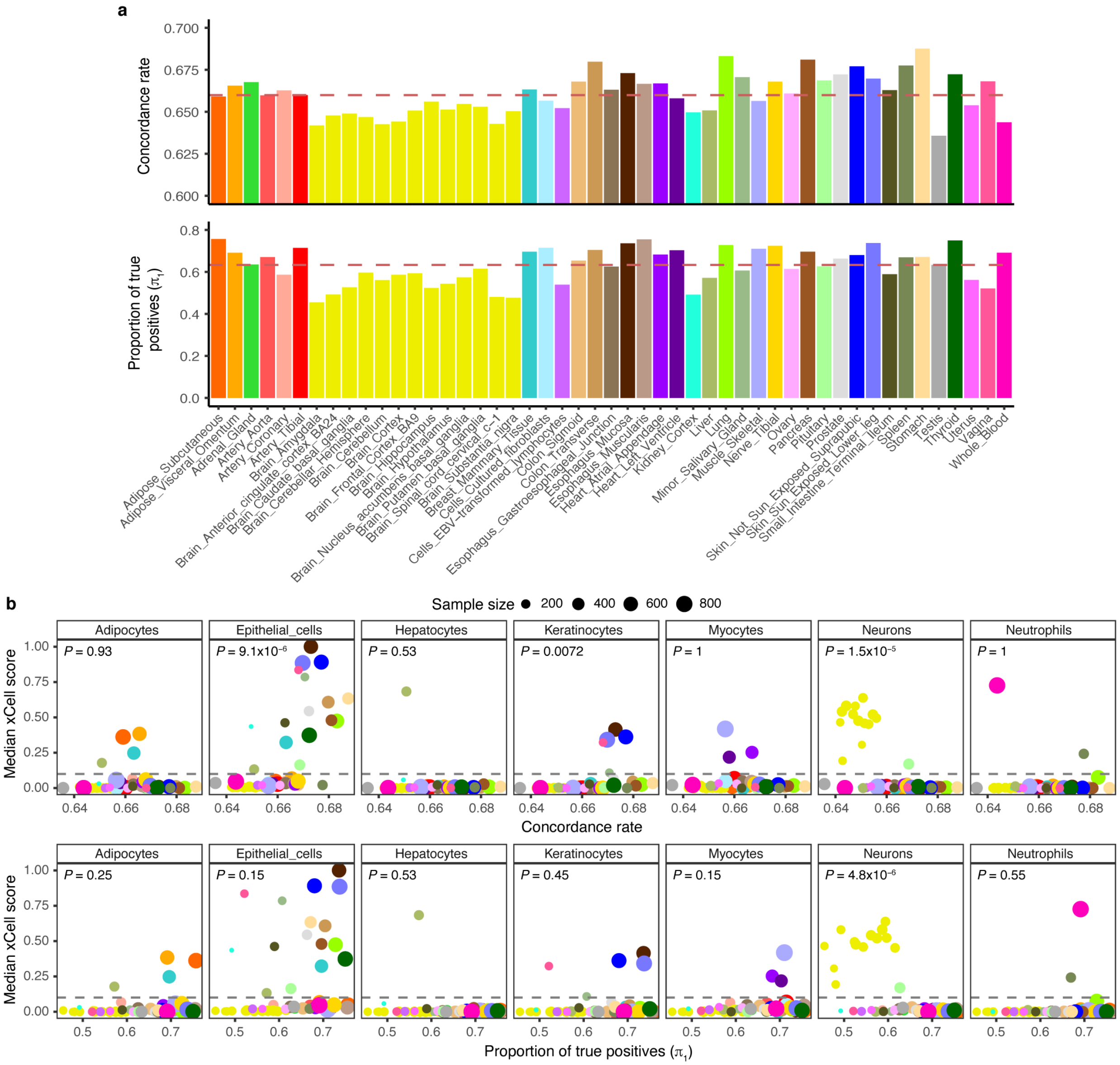

Figure S9. Replication of *cis*-eQTLs in GTEx. a, Replication of *cis*-eQTLs from bronchial epithelium in GTEx v8 using the concordance rate (proportion of gene-variant pairs with the same direction of the effect, upper panel) and proportion of true positives (π_1, lower panel). Red dashed line denotes the median of the respective replication measure. **b**, Relationship between the two replication measures as the function of cell type enrichment of the tissues measured as median enrichment score from xCell for seven cell types: adipocytes, epithelial cells, hepatocytes, keratinocytes, myocytes, neurons, and neutrophils. Grey dashed line denotes median enrichment score > 0.1, which classifies tissues as enriched for the given cell type or not. Wilcoxon rank sum test was used to estimate the difference in replication estimates between tissues enriched or not enriched for the given cell type.

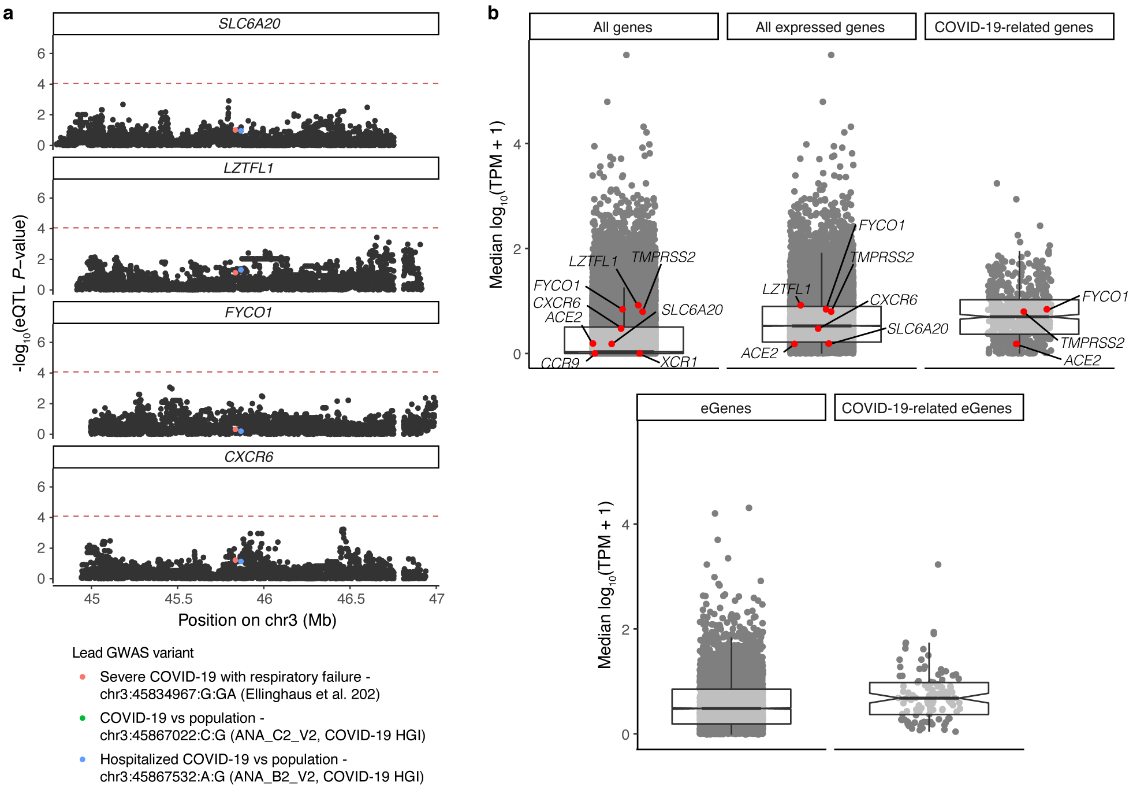

Figure S10. Regulatory genetic effects of the candidate genes in the chr3 cluster associated with COVID-19. a, Regional association plots illustrating no statistically significant regulatory effects on the gene expression levels of *SLC6A20, LZTFL1, FYCO1, CXCR6* in the chr3 locus associated with hospitalized COVID-19 [2] and severe COVID-19 with respiratory failure [3]. Each data point represents a genetic variant in the +/- 1Mb *cis*-window of the transcription start site of the given gene. Red dashed line represents nominal *P*-value = 10^{-4}. *CCR9* and *XCR1* were excluded from eQTL mapping due to low expression in our bronchial epithelium dataset. **b,** Median expression levels of *ACE2*, *TMPRSS6* and the six genes in the chr3 locus associated with COVID-19. Out of the eight genes, we observed that *LZTFL1* that is known to be expressed in the cells of the respiratory epithelium in the lung [3] was among the top 25% of the genes expressed in bronchial epithelium, while other genes are not as highly expressed. The boxes denote the interquartile range, the center line denotes the median, and whiskers denote the interquartile range×1.5.

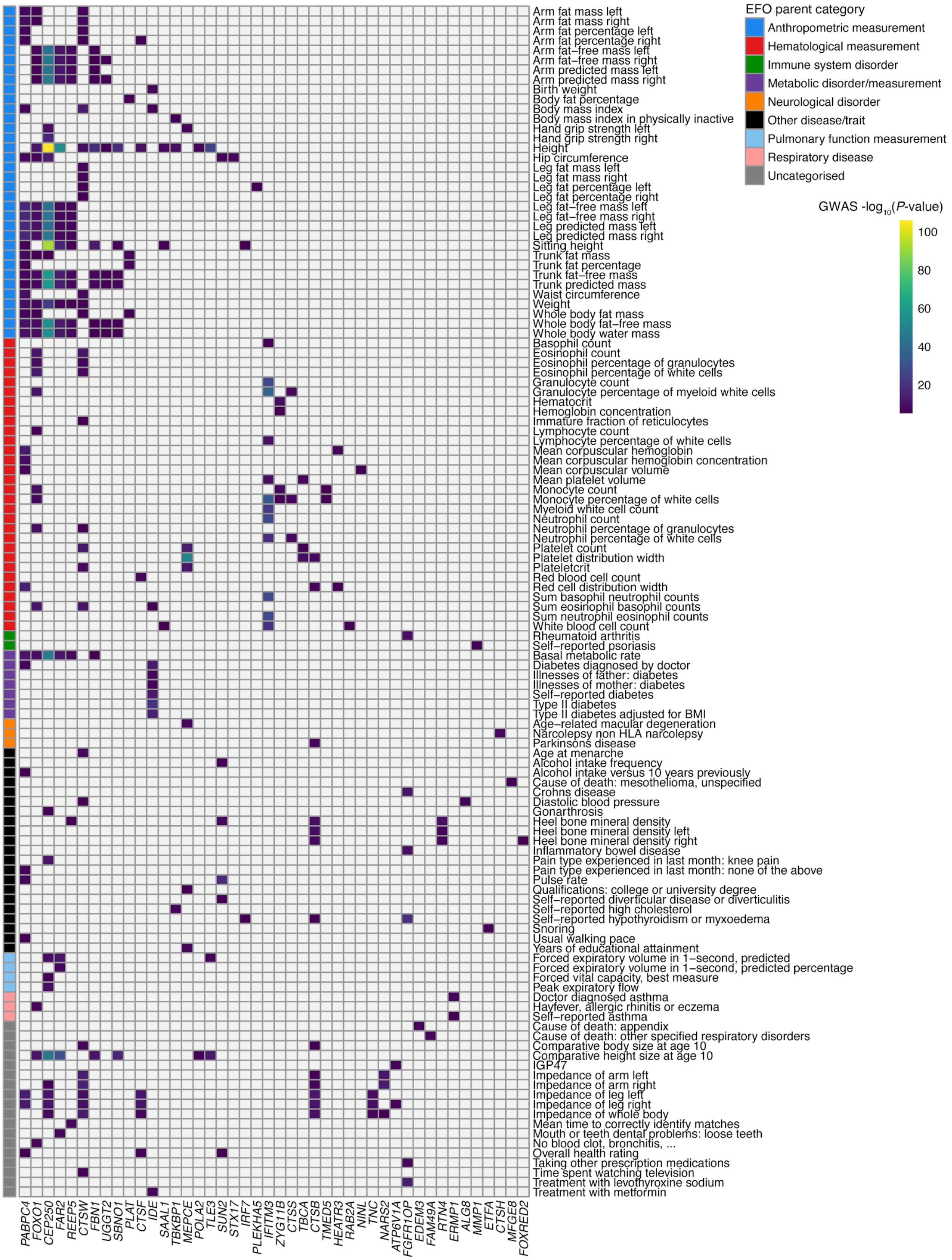

EFO parent category
Anthropometric measurement
Hematological measurement
Immune system disorder
Metabolic disorder/measurement
Neurological disorder
Other disease/trait
Pulmonary function measurement
Respiratory disease
Uncategorised
GWAS -log10(P-value)
100
80
60
40
20

Figure S11. PheWAS associations for the 44 out of 108 lead *cis*-eQTLs associated with COVID-19-related genes with Phenoscanner v2. Heatmap showing the pheWAS associations of the *cis*-eQTLs for COVID-19-related genes. Traits are grouped based on the experimental factor ontology (EFO) terms, and EFO terms with few traits grouped into one group, "Other diseases/traits". Tiles are colored by the $-\log_{10}$(GWAS *P*-value) or are colored grey, if there was no suggestive signal obtained for the given variant and trait (GWAS *P*-value $> 10^{-5}$).

References

1. Mick E, Kamm J, Pisco AO, Ratnasiri K, Babik JM, Calfee CS, et al. Upper airway gene expression differentiates COVID-19 from other acute respiratory illnesses and reveals suppression of innate immune responses by SARS-CoV-2. Preprint at medRxiv https://doi.org/10.1101/2020.05.18.20105171. 2020.
2. COVID-19 Host Genetics Initiative. The COVID-19 Host Genetics Initiative, a global initiative to elucidate the role of host genetic factors in susceptibility and severity of the SARS-CoV-2 virus pandemic. Eur J Hum Genet. 2020;28:715–8.
3. Ellinghaus D, Degenhardt F, Bujanda L, Buti M, Albillos A, Invernizzi P, et al. Genomewide Association Study of Severe Covid-19 with Respiratory Failure. N Engl J Med. 2020.
4. Blanco-Melo D, Nilsson-Payant BE, Liu W-C, Uhl S, Hoagland D, Møller R, et al. Imbalanced Host Response to SARS-CoV-2 Drives Development of COVID-19. Cell. 2020;181:1036-1045.e9.
5. Gordon DE, Jang GM, Bouhaddou M, Xu J, Obernier K, White KM, et al. A SARS-CoV-2 protein interaction map reveals targets for drug repurposing. Nature. 2020;583:459–68.
